# The primary macrophage chemokine, CCL2, is not necessary after a peripheral nerve injury for macrophage recruitment and activation or for conditioning lesion enhanced peripheral regeneration

**DOI:** 10.1186/s12974-022-02497-9

**Published:** 2022-07-12

**Authors:** Aaron D. Talsma, Jon P. Niemi, Joel S. Pachter, Richard E. Zigmond

**Affiliations:** 1 Department of Neurosciences, Case Western Reserve University, 10900 Euclid Avenue, Cleveland, OH 44106-4975 USA; 2 Department of Immunology, University of Connecticut Health Center, Farmington, CT 06030-6125 USA

**Keywords:** Macrophage, CCL2, Regeneration, Axotomy, Neuroimmune, DRG

## Abstract

**Background:**

Peripheral nerve injuries stimulate the regenerative capacity of injured neurons through a neuroimmune phenomenon termed the conditioning lesion (CL) response. This response depends on macrophage accumulation in affected dorsal root ganglia (DRGs) and peripheral nerves. The macrophage chemokine CCL2 is upregulated after injury and is allegedly required for stimulating macrophage recruitment and pro-regenerative signaling through its receptor, CCR2. In these tissues, CCL2 is putatively produced by neurons in the DRG and Schwann cells in the distal nerve.

**Methods:**

*Ccl2*^*fl/fl*^ mice were crossed with Advillin-Cre, P0-Cre, or both to create conditional *Ccl2* knockouts (CKOs) in sensory neurons, Schwann cells, or both to hypothetically remove CCL2 and macrophages from DRGs, nerves or both. CCL2 was localized using Ccl2–RFP^fl/fl^ mice. CCL2–CCR2 signaling was further examined using global *Ccl2* KOs and *Ccr2*^*gfp*^ knock-in/knock-outs. Unilateral sciatic nerve transection was used as the injury model, and at various timepoints, chemokine expression, macrophage accumulation and function, and in vivo regeneration were examined using qPCR, immunohistochemistry, and luxol fast blue staining.

**Results:**

Surprisingly, in all CKOs, DRG *Ccl2* gene expression was decreased, while nerve *Ccl2* was not. CCL2–RFP reporter mice revealed CCL2 expression in several cell types beyond the expected neurons and Schwann cells. Furthermore, macrophage accumulation, myelin clearance, and in vivo regeneration were unaffected in all CKOs, suggesting CCL2 may not be necessary for the CL response. Indeed, *Ccl2* global knockout mice showed normal macrophage accumulation, myelin clearance, and in vivo regeneration, indicating these responses do not require CCL2. CCR2 ligands, *Ccl7* and *Ccl12*, were upregulated after nerve injury and perhaps could compensate for the absence of *Ccl2*. Finally, *Ccr2*^*gfp*^ knock-in/knock-out animals were used to differentiate resident and recruited macrophages in the injured tissues. C*cr2*^*gfp/gfp*^ KOs showed a 50% decrease in macrophages in the distal nerve compared to controls with a relative increase in resident macrophages. In the DRG there was a small but insignificant decrease in macrophages.

**Conclusions:**

CCL2 is not necessary for macrophage accumulation, myelin clearance, and axon regeneration in the peripheral nervous system. Without CCL2, other CCR2 chemokines, resident macrophage proliferation, and CCR2-independent monocyte recruitment can compensate and allow for normal macrophage accumulation.

## Introduction

Extensive research has investigated factors that promote peripheral nerve regeneration, including Wallerian degeneration, activation of neuronal growth programs, changes in Schwann cells, and inflammation. Peripheral axons, presumably due to a combination of these factors, have another remarkable ability: if they are injured a second time, their regeneration rate is greater than after a single injury [1]. This is called the conditioning lesion (CL) response, as the first injury “conditions” axonal growth.

Inflammation, and particularly macrophages, have been implicated in the CL response (e.g., [2]). Macrophages were initially observed in peripheral nerves distal to the injury site (distal nerve, DN; [3]). As professional phagocytes, they purportedly remove inhibitory molecules to permit axonal regeneration. However, macrophages are plastic, capable of creating inflammatory, cytotoxic environments and anti-inflammatory, pro-regenerative environments [4, 5]. Within the DN, macrophages promote growth factor production, which may aid regeneration [6, 7]. Macrophages were also discovered in sensory (i.e., dorsal root ganglia; DRGs) and sympathetic ganglia after peripheral nerve injury [8, 9], and DRG macrophages can produce CL-like regeneration enhancement in the dorsal root [10]. These macrophages were hypothesized to produce cytokines, which directly or indirectly stimulate DRG somata. Thus, there are two macrophage populations, which could be critical for the CL response: DRG macrophages and DN macrophages.

Within these two populations there are at least two subpopulations: resident and recruited macrophages. The presence of recruited macrophages in the DRG [11, 12] and DN [13, 14] implies the presence of a recruitment signal. CCR2 is the primary macrophage chemokine receptor responsible for their chemotaxis into tissues [15, 16], and it seems necessary for recruiting macrophages to the injured DRGs and DN. Furthermore, when these macrophages are absent, as in the *Ccr2* knockout (KO) mouse, the CL response is largely abolished in culture assays [12]. CCL2 is the primary ligand for CCR2 [17, 18], and it recruits macrophages to a variety of injury and infection sites. It is rapidly upregulated in DRGs and DNs after a peripheral nerve injury, suggesting it functions in the injury response [19, 20]. Indeed, Kwon et al. [21] reported that CCL2 is required for the CL response. In *Ccl2* KO mice, they found no macrophage recruitment to DRGs after sciatic nerve injury. Furthermore, they reported that CCL2 is required to stimulate pro-regenerative signaling by macrophages in co-culture assays, suggesting dual critical functions for CCL2 in recruitment and regeneration.

Given the data suggesting that recruited macrophages are necessary for the CL response and that CCL2 is required to recruit macrophages, we sought to create site-specific *Ccl2* KOs to prevent recruitment to either DRGs or DNs. Previous reports claimed that CCL2 is only expressed by neurons in DRGs [19, 22, 23], and is primarily expressed by Schwann cells in peripheral nerves [24, 25]. We used two Cre drivers to target these cell populations: Advillin-Cre, which expresses in most sensory neurons [26], and P0-Cre, which expresses in Schwann cells [27]. Crossing these drivers to CCL2^fl/fl^ mice [28], we generated three conditional *Ccl2* KOs (*Ccl2* CKOs): Adv-Cre; CCL2^fl/fl^ mice (ACKOs) to reduce DRG CCL2 expression and macrophage recruitment; P0-Cre; CCL2^fl/fl^ mice (PCKOs) to reduce DN CCL2 expression and macrophage recruitment; and a double CKO (DCKO) containing both Cre drivers. Instead of demonstrating the distinct functions of DRG- and DN-recruited macrophages, we show that assertions about CCL2’s function in a sciatic nerve injury model have to be reconsidered.

## Materials and methods

### Mice

**Fig. 1:**
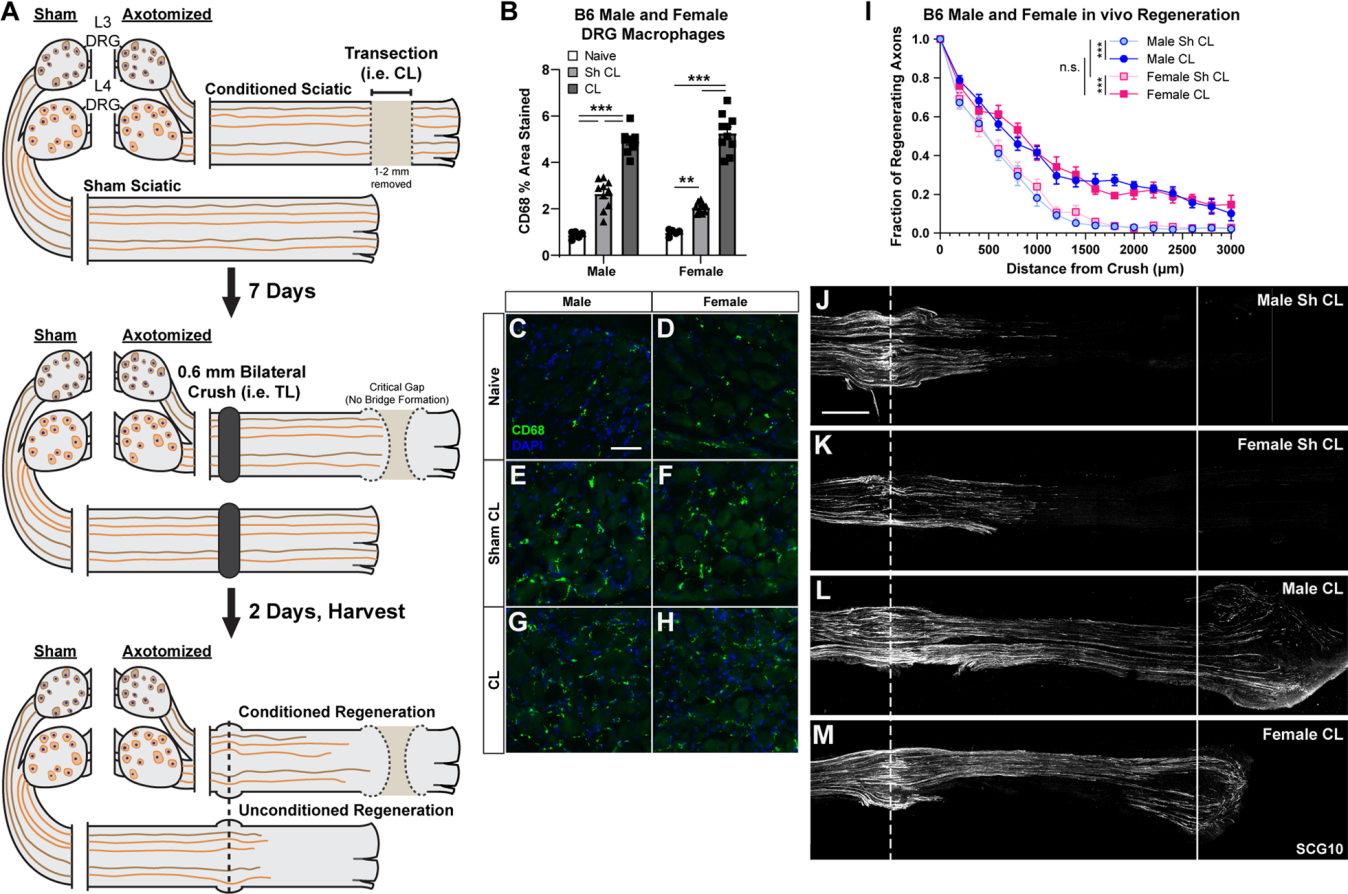
Male and female B6 (WT) mice have indistinguishable DRG macrophage accumulation and activation, and conditioned and unconditioned in vivo regeneration. **A** Diagram of our in vivo conditioning lesion (CL) and regeneration paradigm. **B** Macrophage quantification in Naïve, 2 day post-crush (Sh CL), and 9 day post-transection plus 2 day post-crush (CL) DRGs from male and female mice. Macrophages were quantified as the percent area stained by anti-CD68. Means were compared with a two-way ANOVA followed by Tukey’s post-hoc tests. **C**–**H** Representative images of macrophages in the cell body area of L4 DRGs from the indicated sex and injury condition. Scale bar is 50 μm. **I** Axon regeneration expressed as the fraction of axons relative to the crush site every 200 µm. Each point is the mean fraction ± SEM. Pairs of curves were compared using non-linear regression assuming one phase decay, initial *Y* = 1, and the plateau = 0 and significance was determined by comparing the decay constants of the fitted curves. **J**–**M** Representative images of regenerating axons stained for SCG10, a marker of regenerating sensory axons. Unconditioned growth (**J**, **K**), representing neuron intrinsic regeneration rate, and conditioned growth (**L**, **M**) were the same between sexes. Conditioned growth was increased compared to unconditioned growth in both sexes. The dotted line indicates the center of the crush site which was considered to be 500 μm wide, and the solid line is 3000 μm from the crush site. Scale bar is 500 μm. n = 10 for all groups and * indicates significance differences (**p < 0.01, ***p < 0.001) between indicated means

Eight-to-twenty-four-week-old age matched mice were used for all studies. As shown in Fig. 1B–M, no sexual dimorphism was found after sciatic nerve transection in macrophage accumulation in either the DRG or in the CL response. Therefore, males and females were used in approximately equal numbers for all groups in all experiments. Mice were housed under 12:12 light:dark cycle with ad libitum access to food and water. The conditional knockout genotypes used for this study were Advillin-Cre; CCL2^fl/fl^, P0-Cre; CCL2^fl/fl^, and Advillin-Cre; P0-Cre; CCL2^fl/fl^, with CCL2^fl/fl^ used as the control. These strains were bred by our lab from CCL2^fl/fl^ (from Joel Pachter; Ge et al., [28]), Advillin-Cre (from Paul Heppenstall, European Molecular Biology Lab, Monterotondo, Roma, Italy; [26]), and P0-Cre (Jackson Laboratories, Bar Harbor, ME, USA; # 017927) strains. The CCL2–RFP^fl/fl^ mice (Jackson Labs; #016849; [29]) were bred with Advillin-Cre and P0-Cre strains to create the CCL2–RFP DCKO. For the *Ccl2* global KO experiments, we used WT controls (C57BL/6J; Jackson Labs; #000664) and *Ccl2*^*−/−*^ (Jackson Labs; #004434) strains [30]. For measuring the contribution of CCR2 to macrophages accumulation, we bred *Ccr2*^*gfp/gfp*^ Knock-in (KI)/KO (Jackson Labs; #027619) animals with C57BL6/J to create heterozygous *Ccr2*^*gfp/*+^ controls or with themselves to make *Ccr2*^*gfp/gfp*^ (i.e., *Ccr2* KOs) [31].

### Surgeries

The following surgical procedures were approved by the Case Western Reserve University Institutional Animal Care and Use Committee.

#### Sciatic nerve transection

Mice were anesthetized with isoflurane anesthesia. A small incision was made in the thigh distal to the sciatic notch, and the sciatic nerves were exposed. The nerve was transected unilaterally on the right side in the proximal thigh near the greater trochanter, and a piece of nerve, the width of #55 forceps (Fine Science Tools, Foster City, CA, USA; 11254-20), was removed to impair regeneration. On the left side, the nerve was only exposed as a control. The wounds were closed with a wound clip. For analgesia, animals were given bupivacaine subcutaneously at the incision and carprofen subcutaneously at the scruff. The animals were allowed to recover for 1, 2 or 7 days before they were sacrificed by CO_2_ inhalation. L3 and L4 DRGs and the sciatic nerves were removed for immunofluorescence or molecular biological analysis.

#### In vivo conditioning lesion

Mice were anesthetized with isoflurane, and a small incision was made in the mid-thigh. On the right side, the sciatic nerve was exposed and transected at the trifurcation, and a forceps-width of nerve was removed. On the left, the trifurcation was only exposed as a control. Wounds were closed with a wound clip, and the mice were allowed to recover for 7 days. At the end of this period, mice were again anesthetized. The original incision was reopened, and the sciatic nerve was exposed at the level of the greater trochanter of the femur. The sciatic nerves were crushed bilaterally using ultrafine hemostats (Fine Science Tools; 13006-12) for 45 s just distal to the greater trochanter. The wounds were closed with wound clips. The mice recovered for 2 days before they were sacrificed by CO_2_ inhalation. The sciatic nerves were removed, pinned in a 35 mm petri dish with dental wax in the bottom so they were straight, and fixed overnight in 4% paraformaldehyde in PBS. After both surgeries, animals were given bupivacaine subcutaneously at the incision and carprofen subcutaneously at the scruff.

### RT-qPCR

One or two days after sciatic nerve transection, conditional KO animals were sacrificed. The L3 and L4 DRGs from each side (i.e., axotomized and sham sides) were removed, pooled and flash frozen with liquid nitrogen. The right sciatic nerve distal to the transection and an equivalent length of the left sciatic nerve were removed and flash frozen. The tissue was stored on dry ice or at − 80 °C. For the DRGs, RNA was extracted using the RNAqueous Micro kit (Thermo Fisher Scientific, Waltham, MA, USA; AM1931). For the sciatic nerves, RNA was extracted using the TRIzol Plus RNA Purification kit (Thermo Fisher; 12183-555) as described in the Thermo Fisher protocol. RNA was made into cDNA using the qScript cDNA Synthesis kit (Quanta Bio, Beverly, MA, USA; 95047-100). qPCR was done in triplicate in an Applied Biosystems Step-One Plus (Thermo Fisher; 4376600) using pre-validated Taqman expression assays (Thermo Fisher; 4331182) for *Gapdh* (Mm99999915_g1), *Ccl2* (Mm00441242_m1) *Ccl7* (Mm00443113_m1), and *Ccl12* (Mm01617100_m1). Relative expression was quantified using the ddCT method with *Gapdh* as the housekeeping gene.

### Luxol fast blue (LFB) for myelin visualization

Sciatic nerves were drop fixed in 4% paraformaldehyde (PFA) in phosphate-buffered saline (PBS), washed, cryoprotected in 30% sucrose in PBS, mounted in Tissue-Tek O.C.T. compound (Electron Microscopy Sciences, Hatfield, PA, USA; 62550-01), sectioned with a cryostat and direct mounted onto Superfrost Plus slides (Thermo Fisher; 12-550-15). LFB staining was done on 20 μm sections. Slides were rehydrated in water for 5 min then moved to 35% ethanol for 5 min then to 70% for 5 min before being placed in sealed slide containers with filtered 0.1% LFB solution (Electron Microscopy Sciences; 26681-01) and incubated at 60–65 °C overnight. Slides were rinsed briefly by dipping first in 95% ethanol and then in distilled water. Slides were destained by incubating in 0.05% lithium carbonate (w/v) in ddH_2_O for 30 s and then immediately quenched by rinsing and incubating in 70% ethanol for 5 min. Slides were dehydrated by incubating in 95% ethanol and then 100% ethanol for 5 min each, soaked in xylenes for 5 min, and then cover-slipped with VectaMount Mounting Medium (Vector Laboratories, Burlingame, CA, USA; H-5000). Slides were imaged blinded at ×20 magnification under a light microscope with constant settings. Three images were taken per nerve or six total per animal. Images were quantified blind as the LFB stained area as a percentage of the section area. Percent LFB area of the three images per nerve were averaged to give a measure of myelin clearance per nerve per animal.

### Immunofluorescence and imaging

Sciatic nerves and L4 DRGs were harvested, cleaned briefly, and drop fixed in 4% PFA overnight before cryoprotection in 30% sucrose in PBS for at least 2 days. Pairs of sham and manipulated DRGs or sciatic nerves from each animal were embedded in Tissue-Tek O.C.T compound (Electron Microscopy Sciences), sectioned at 10, 20 and 40 μm using a cryostat, and direct mounted onto Superfrost Plus slides (Thermo Fisher; 12-550-15). Tissue was stained on the slides, using a PAP pen ring to contain the staining solutions. Sections were incubated with primary antibodies overnight at 4 °C, and subsequently incubated in secondary antibodies for either 1 h, for 10 μm sections, or 2 h, for 20 and 40 μm sections, at room temperature. The following primary antibodies were used at the following dilutions in blocking buffer: rat anti-CD68 1:200 (Bio-Rad Laboratories, Hercules, CA, USA; MCA1957), rabbit anti-Iba1 1:500 (Fujifilm Wako Chemicals, Richmond, VA, USA; 019-19741), rabbit anti-SCG10 1:4000 (Novus Biologicals, Centennial, CO, USA; NBP1-49461), rabbit anti-RFP 1:800 (Rockland Immunochemicals, Limerick, PA, USA; 600-401-379), rat anti-mCherry 1:1000 (Thermo Fisher; M11217), rat anti-F4/80 1:1000 (Bio-Rad; MCA497), rabbit anti-ΒIII-tubulin 1:1000 (Abcam, Waltham, MA,USA; ab18207) and sheep anti-green fluorescent protein (GFP; Bio-Rad; 4745-1051). The following secondary antibodies were used at a 1:400 dilution in blocking buffer: Cy3 donkey anti-rat (Jackson ImmunoResearch, West Grove, PA, USA; 712-165-153), AF555 donkey anti-rat (Abcam; ab150154), AF594 donkey anti-rat (Jackson ImmunoResearch; 712-585-153), AF594 donkey anti-rabbit (Jackson ImmunoResearch; 711-585-152), AF488 donkey anti-rabbit (Jackson ImmunoResearch; 711-545-152), AF647 donkey anti-rat (Jackson ImmunoResearch; 712-605-153), and AF488 donkey anti-sheep (Jackson ImmunoResearch; 713-546-147). DAPI 1:1000 in PBS (Thermo Fisher; D1306) was used to label cell nuclei. Ten μm sections were stained to examine macrophages in the *Ccl2* CKOs and *Ccl2* KOs, and CCL2-RFP localization in the sciatic nerve (Figs. 3, 4, 8, 9). Twenty μm sections were stained to examine CCL2–RFP DRG localization in the Ccl2–RFP^fl/fl^ controls and Ccl2–RFP DCKOs (Fig. 7). Forty μm sections were stained to examine in vivo axon regeneration (Figs. 5, 10).

To quantify macrophage accumulation in the DRG and sciatic nerve (Figs. 1, 3, 4, 9), images were captured at ×25 magnification using SimplePCI software (Hamamatsu) and constant exposure settings. Three images per nerve or DRG were captured and used for quantification giving a total of 12 images per animal. The area of the section that was stained is expressed as a percentage of the total area examined. The three values from each tissue and condition (e.g. sham DRG) were averaged to give a single value for each tissue and condition in each animal. Images to quantify nerve regeneration and CCL2–RFP localization were taken using a Zeiss LSM 800 with constant settings (laser powers, scan speed, resolution and PMT voltages; Figs. 1, 5, 7, 8, 10). All confocal stacks were run through a 3 by 3 median filter to remove noise before they were maximum projected for quantification. The investigator was blinded to the groups before quantification. Quantification of nerve regeneration is described below. Images were quantified using ImageJ software (1.53).

### In vivo conditioning lesion regeneration analyses

Nerve regeneration was visualized by staining 40 μm nerve sections with an antibody against SCG10. The most straightforward way to quantify these images would be to identify and measure the length from the crush of each regenerating axon. However, there were several challenges in quantifying these images. The first major problem is that the sections are never exactly parallel to the growth of the regenerating axons. This means that along the length of the nerve some axons are leaving the section and others are entering. Second, there are many regenerating axons within a 40 μm section, and they are often packed tightly, particularly within and near the test lesion (i.e. Crush Site). These facts mean that it is extremely rare to be able to identify single axons or even their tips, and thus, measuring all regenerating axons individually was not possible. Therefore, we devised another indirect method for quantifying regeneration.

This quantification method relies on five assumptions. (1) The number of axons leaving and entering a section at any particular point is equal. (2) The axons located within the crush site are the maximum number of axons that can regenerate within the section and do not terminate within the crush site. (3) The diameter of a particular regenerating axon is constant along its length. (4) All axonal subtypes of differing calibers have the same capacity to regenerate, or expressed mathematically, the fraction of axons of a particular caliber is constant along the length of the nerve. (5) Each section is approximately straight and has the same width (i.e. diameter) at the proximal and distal ends, so that the number of axons that could grow is the same in the proximal and distal ends of the segment. This assumption was met by pinning the nerve straight during fixation. Using the first 4 assumptions, we can show that the number of SCG10 positive pixels can be counted to measure both the fraction of regenerating axons at a given distance from the crush as well as the average length of all regenerating axons.

From assumptions 3 and 4, let there be *k* axon subtypes, where *c*_*i*_ is the caliber of each subtype, *F*_*i*_ is the fraction of each subtype, and where $k$, ${c}_{i}$, and ${F}_{i}$ are constants. Let ${n}_{i}(x)$ be the number of axons of caliber ${c}_{i}$ at a distance *x* from the crush site and ${n}_{T}(x)$ be the total number of axons at a distance *x*. By these definitions, ${F}_{i} = {n}_{i}/{n}_{T}$ at every distance. The total width of all axons of caliber ${c}_{i}$ is ${w}_{i}={c}_{i}*{n}_{i}$ and the total width of all axons is ${w}_{T}= \sum_{i=1}^{k}{c}_{i}* {n}_{i}$ at given distance. Substituting for ${n}_{i}$, ${w}_{i}={c}_{i}*{F}_{i}*{n}_{T}(x)$, and ${w}_{T}= \sum_{i=1}^{k}{c}_{i}* {F}_{i}*{n}_{T}(x)$ or ${w}_{T}= {n}_{T}\left(x\right)*\sum_{i=1}^{k}{c}_{i}* {F}_{i}$. Thus, the fractional width, ${W}_{i}$, of all ${c}_{i}$ caliber axons at a given distance is

$$W_{i} \left( x \right) = {\raise0.7ex\hbox{${w_{i} \left( x \right)}$} \!\mathord{\left/ {\vphantom {{w_{i} \left( x \right)} {w_{T} \left( x \right)}}}\right.\kern-\nulldelimiterspace} \!\lower0.7ex\hbox{${w_{T} \left( x \right)}$}} = \frac{{c_{i} *F_{i} *n_{T} \left( x \right)}}{{n_{T} \left( x \right)*\mathop \sum \nolimits_{i = 1}^{k} c_{i} * F_{i} }} = \frac{{c_{i} *F_{i} }}{{\mathop \sum \nolimits_{i = 1}^{k} c_{i} * F_{i} }}.$$

Since ${c}_{i}$ and ${F}_{i}$ are constants, ${W}_{i}$ is also a constant. ${n}_{i}(x)$ can be expressed in terms of axon width: ${n}_{i}\left(x\right)= \frac{{w}_{i}(x)}{{c}_{i}}=\frac{{W}_{i}*{w}_{T}(x)}{{c}_{i}}$ and thus

$$n_{T} \left( x \right) = \mathop \sum \limits_{i = 1}^{k} n_{i} \left( x \right) = \mathop \sum \limits_{i = 1}^{k} \frac{{W_{i} *w_{T} \left( x \right)}}{{c_{i} }} = w_{T} \left( x \right)*\mathop \sum \limits_{i = 1}^{k} \frac{{W_{i} }}{{c_{i} }}.$$

This relationship is critical, because it means that the total number of axons at a given distance is directly proportional to the total width of all axons multiplied by a constant ratio, and furthermore, the total width of all SCG10 positive axons can easily be measured using FIJI.

#### Fractional outgrowth

To measure the widths of all axons at distances along the regenerating nerve, we wrote an ImageJ macro. The macro uses rectangular ROIs placed on the crush site and at various distances along the nerve to quantify the SCG10 staining. The user sets a threshold for positive SCG10 staining and then the macro records the Percent Area of positive staining, or PAS, along with the length (or *X* length) and width (or *Y* length) of the ROI among other parameters. By the definition of percent area, $$\mathrm{PAS}=\frac{\mathrm{Positive} \,\mathrm{Pixels}}{\mathrm{ROI} \mathrm{Area}}*100$$, or for a rectangular ROI, $$\mathrm{PAS}=\frac{\mathrm{Pixel}\, \mathrm{number}}{X*Y}*100$$, so $$\mathrm{Positive} \,\mathrm{Pixels}=\frac{\mathrm{PAS}*X*Y}{100}$$. However, Positive Pixels is proportional to an area of axons, and we need to know the total width to find the number of axons.

From assumption 2, because we assume that no axons terminate within the crush site, the length of all axons should be a constant, *L* or the X dimension of the ROI, and the total width of all axons (stacked in the Y dimension) should be constant along the length of the crush site, and we will call the width: ${w}_{T}\left(0\right)$. From above, the total area of axons in pixels within the crush is the number of Positive Pixels and thus, $\mathrm{Positive} \,\mathrm{Pixels}=L*{w}_{T}\left(0\right)$. Therefore, ${w}_{T}\left(0\right)=\mathrm{Positive} \,\mathrm{Pixels}/L=\frac{\mathrm{PAS}*Y}{100}$ and this is also proportional to ${n}_{T}(0)$ as demonstrated above.

To determine ${w}_{T}(x)$ at other points along the nerve is more difficult. If the ROI were infinitesimally small, or in the case of digital images, a pixel wide, then we would know that any axon in that ROI has the length of a pixel. With a constant axon length, ${w}_{T}(x)$ can be determined as above. However, imaging is inherently noisy and using single pixel wide ROIs would increase the variability of the quantification. Instead, we used a running average of the axon widths centered on each distance of interest. This was measured in a similar way to the axon width in the crush site. If we make an ROI of length *L*, then it can be considered to be *L* sequential ROIs of width 1. As above, ${w}_{T}\left(x\right)=\mathrm{Positive} \,\mathrm{Pixels}/X=\mathrm{Positive} \,\mathrm{Pixels}$, since *X* = 1. The average ${w}_{T}\left(x\right)$ is the sum of ${w}_{T}\left(x\right)$ for all *L* sequential ROIs divided by the number of terms, or *L*. Since the sum of all the widths is the same as the total number of pixels within the length *L* ROI, $\mathrm{average}\, {w}_{T}\left(x\right)=\mathrm{Positive} \,\mathrm{Pixels}/L=\frac{\mathrm{PAS}*Y}{100}$ which is proportional to ${n}_{T}(x)$.

Finally, the fraction of axons regenerating to a given distance is $F\left(x\right)={n}_{T}(x)/{n}_{T}(0)$. Expressing the number of axons in terms of their widths:

$$F\left(x\right)=\frac{{\mathrm{average}\, w}_{T}\left(x\right)*\sum_{i=1}^{k}\frac{{W}_{i}}{{c}_{i}}}{{w}_{T}\left(0\right)*\sum_{i=1}^{k}\frac{{W}_{i}}{{c}_{i}}}=\frac{\mathrm{average}\, {w}_{T}\left(x\right)}{{w}_{T}\left(0\right)}=\frac{{\mathrm{PAS}}_{\mathrm{ROI}}*{Y}_{\mathrm{ROI}}/100}{{\mathrm{PAS}}_{\mathrm{crush}}*{Y}_{\mathrm{crush}}/100}$$

To quantify the regeneration images, we wrote a FIJI macro to use this relationship. It requires the user to place a rectangular ROI on the crush site and then trace the upper and lower borders of the nerve distal to the crush. It will then place ROIs at intervals distal to the crush to measure outgrowth. The macro can be adjusted to use any width for the crush site, any distance interval for the outgrowth ROIs, and any width for the outgrowth ROIs. For this paper, the crush width was 500 μm, the interval was 100 μm, and the ROI width was 25 μm. Images were blinded in pairs so that the sham operated nerve and the conditioned nerve were quantified together, which allowed us to use the sham fluorescence intensity as a reference for the conditioned nerve on the same slide.

#### Average axon length

The best measure of regeneration is the average axon length, which is calculated from the sum of all axon lengths divided by the number of axons. However, since individual axon lengths could not be reliably measured, we needed to indirectly derive their length from the area of SCG10 staining. First, the total area, ${A}_{i}$, of axons of caliber ${c}_{i}$ in a given region is the sum of width of each axon times the length of each axon, ${l}_{a}$, or ${A}_{i}={\sum }_{a=1}^{{n}_{i}}{l}_{a}*{c}_{i}$. Since we cannot measure the length of axons within an arbitrary area, we will constrain the length of the area to be infinitely small (i.e. 1 pixel). Thus, if an axon is contained in this area, then we know its length to be dx and thus

$${A}_{i}={\sum }_{a=1}^{{n}_{i}}{c}_{i}*\mathrm{d}x= {{n}_{i}(x)*c}_{i}*\mathrm{d}x= {w}_{i}(x)*\mathrm{d}x$$

Since the axons are all the same caliber, their summed length is their area divided by their caliber or ${L}_{i}\left(x\right)={{A}_{i}(x)}/{{c}_{i}}=\frac{{w}_{i}(x)}{{c}_{i}}*{\text{d}}x$. Substituting ${W}_{i}*{w}_{T}(x)$ for ${w}_{i}(x)$ and summing the lengths of axons of all calibers gives

$$L(x)={\sum }_{i=1}^{k}\frac{{W}_{i}*{w}_{T}(x)}{{c}_{i}}*\mathrm{d}x$$

for the length of all axons at distance *x*. Thus, to find the total length of all axons we simply need to sum each infinitesimal length along the entire length of nerve:

$${L}_{\mathrm{Total}}=\sum_{x=1}^{\infty }{\sum }_{i=1}^{k}\frac{{W}_{i}*{w}_{T}(x)}{{c}_{i}}*\mathrm{d}x=\sum_{x=1}^{\infty }{w}_{T}\left(x\right)*\mathrm{d}x*{\sum }_{i=1}^{k}\frac{{W}_{i}}{{c}_{i}}$$

Using this relationship for total axon length, and the relationship above for the total number of axons in the crush site, which we assume to be the total number of axons, we can calculate the average axon regeneration length:

$$\mathrm{Avg} \mathrm{Length}= \frac{\mathrm{total} \,\mathrm{length}\, \mathrm{of}\, \mathrm{all}\, \mathrm{axons}}{\mathrm{total} \,\mathrm{number} \,\mathrm{of} \,\mathrm{axons}}=\frac{\sum_{x=1}^{\infty }{w}_{T}\left(x\right)*\mathrm{d}x*{\sum }_{i=1}^{k}\frac{{W}_{i}}{{c}_{i}}}{{w}_{T}\left(0\right)*\sum_{i=1}^{k}\frac{{W}_{i}}{{c}_{i}}}=\frac{\sum_{x=1}^{\infty }{w}_{T}\left(x\right)*\mathrm{d}x}{{w}_{T}\left(0\right)}$$

As described above, the total area of axons within an ROI is the total number of positive pixels: $$\mathrm{Positive}\, \mathrm{Pixels}=\frac{\mathrm{PAS}*X*Y}{100}$$ and thus the total width of the axons is $${\mathrm{Positive}\, \mathrm{Pixels}}/{X}=\frac{\mathrm{PAS}*Y}{100}={w}_{T}(x)$$. This is the true length of the axons, since all the axons in these ROIs are the same length: 1 pixel. $$\mathrm{d}x$$ is a constant and equal to the scale in microns of the pixels. Finally, $${w}_{T}\left(0\right)=\mathrm{Positive} \,\mathrm{Pixels}/{L}_{\mathrm{crush}}=\frac{{\mathrm{PAS}}_{\mathrm{crush}}*{Y}_{\mathrm{crush}}}{100}$$ as shown above. Substituting these values in

$$\mathrm{Avg} \,\mathrm{Axon}\, \mathrm{Regen} \,\mathrm{Length}=\frac{\sum_{x=1}^{\infty }{w}_{T}\left(x\right)*\mathrm{d}x}{{w}_{T}\left(0\right)}= \frac{\mathrm{scale}*\sum_{x=1}^{\infty }\frac{\mathrm{PAS}(x)*Y(x)}{100}}{\frac{{\mathrm{PAS}}_{\mathrm{crush}}*{Y}_{\mathrm{crush}}}{100}}$$

To measure these values, we expanded the macro used to find the fractional outgrowth. After it collects the values for outgrowth at various distances, it then draws pixel wide ROIs between the upper and lower bounds until one of them ends. It records the percent area stained and the dimensions of all these ROIs. We then created an Excel macro to pull the relevant values and plug them into the equation above to calculate the average axon length for each nerve. Because the macro performs both quantifications at once, the crush site was 500 μm, and the images were blinded and quantified in pairs.

While this analysis theoretically measures the exact average regeneration length, there are two caveats to its practical application. First, the analysis assumes that pixels can be counted along an infinite length of nerve. This is obviously not possible as the nerve segments are finite which implies an underestimate of average regeneration. However, this underestimate is empirically small. In long nerve segments, only a few of the longest axons terminate beyond 3000 μm from the crush, and when the analysis was constrained to a sum ending at 3000 μm, the decrease in the average length value was less than a micron. Second, the analyzed images are created from a maximum projection of a *Z*-stack. If two axons overlap at all, some of the pixels representing those axons will be lost in the projection. This again would cause a small underestimate but should be comparable across all sections. Thus, this analysis gives an approximate value for average axon regeneration length that approaches the true value.

### Image analysis and presentation

**Fig. 2:**
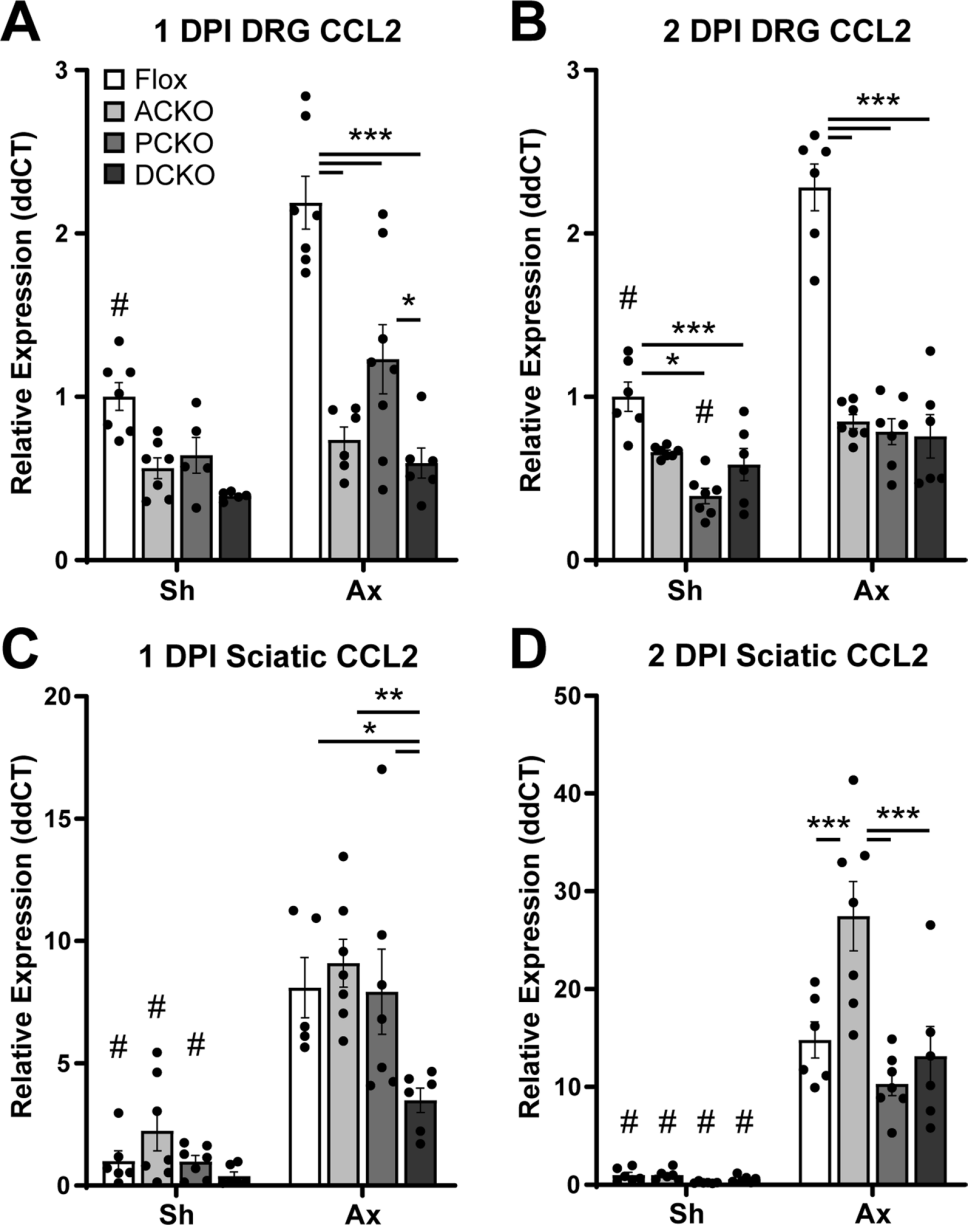
*Ccl2* expression is decreased in the DRG but not the sciatic nerve in the Cre dependent conditional knockouts. **A**, **B** *Ccl2* is upregulated 1 day (**A**) and 2 days (**B**) after injury in Flox control DRGs but not in any of the CKOs. **C**, **D** *Ccl2* is upregulated in the distal sciatic nerves of all genotypes 1 day (**C**) and 2 days (**D**) after injury. # Indicates a significant (*p* < 0.05) difference between sham (Sh) and axotomized (Ax) within a genotype and * indicates a significant difference between indicated genotypes within an injury condition (*p < 0.05, **p < 0.01, ***p < 0.001)

**Fig. 3:**
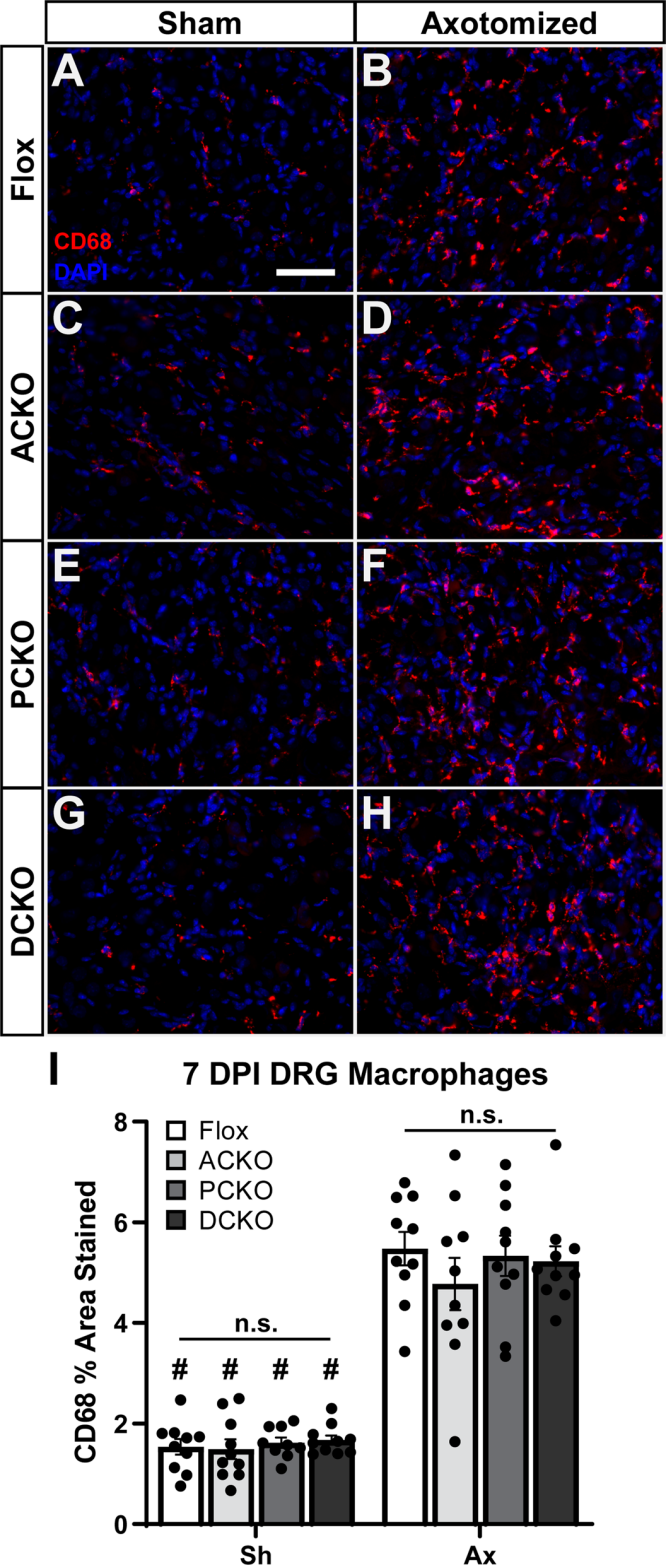
DRG macrophage accumulation and activation is unaffected in the DRG-targeted ACKOs and DCKOs 7 days after injury. **A**–**H** Representative images showing macrophages marked by anti-CD68 in L4 DRGs ipsilateral (**B**, **D**, **F**, **H**) and contralateral (**A**, **C**, **E**, **G**) to a sciatic nerve transection. All genotypes have an increase in macrophages after injury (**A** vs. **B**; **C** vs. **D**; **E** vs. **F**; and **G** vs. **H**) and no differences in macrophages among genotypes either before (**A**, **C**, **E**, **G**) or after (**B**, **D**, **F**, **H**) injury. Scale bar is 50 μm. **I** Macrophages were quantified as the percent area stained by anti-CD68 and plotted as mean ± SEM, *n* = 10 per group. # Indicates a significant (*p* < 0.05) difference between Sh and Ax within a genotype

All images were blinded for quantification. For each immunofluorescence experiment, “no primary” staining controls were run and images of the no primary control slides were used to choose a minimum threshold for the experiment. For quantification of macrophages in the *Ccl2* CKOs and *Ccl2* KOs (Figs. 2, 3, 8), thresholds were set independently for all images and the pixels at or above the threshold were used to measure the percent area stained value for each image. For quantification of CCL2–RFP^+^ cells, no-primary controls were used to determine the minimum threshold, signal below the threshold was removed, and then RFP signals associated with a DAPI-stained nucleus were counted (Figs. 6, 7). For quantification of SCG10 signal, thresholds were set for pairs of images as described in the in vivo Conditioning Lesion Regeneration Analysis section.

Representative images were adjusted to best represent the quantification. Background was removed near to the level of the threshold for the analysis, leaving 5–10 more grey values than the threshold. The same grey level was used to remove background from all the images representing a single experiment. Images were then brightened to best show the positive staining. These steps were necessary, because there was variability in the average staining intensity among slides within the same staining run.

### Statistics and experimental design

Statistical tests were performed in Prism 9 (GraphPad, San Diego, CA). All bar graphs show the mean ± SEM. Data were assumed to be sampled from a normal distribution, and means were compared using a two-way ANOVA with Tukey’s multiple comparisons post hoc tests, *α* = 0.05. Only comparisons within genotypes and within injury conditions were considered relevant and reported. Other comparisons (e.g. Flox Sh vs. ACKO Ax or PCKO Sh vs. ACKO Ax) are not meaningful and were not reported, although they were calculated in the post-hoc test. For each experiment approximately equal numbers of male and female mice were used. At least three animals of each sex were slated for an experimental group when planning, depending on the availability of the genotype, and four of each sex was considered ideal in case a sample was lost. If a cage slated for an experiment contained extra mice those mice were added to their corresponding experimental group. Each animal was considered to be an experimental unit. Accordingly, the *n* for the following experiments is 6–10, and the *n* for each sex is 3–5.

For the qPCR experiments, six animals per genotype per timepoint (three males and three females) were planned to be used and extra animals in the selected cages were included. However, there were several problems when extracting RNA and some samples were lost due to contamination and poor yields. In addition, the amplification results, especially for the sciatic nerves, had a high degree of variability. We used ROUT (*Q* = 0.5%) in Prism to conservatively remove outliers. The resulting n for the qPCR experiments are in Table 1 with the number excluded in parentheses.

**Table 1:** *n* for the qPCR experiments

	1 DPI DRG		2 DPI DRG		1 DPI Sci		2 DPI Sci	
CCL2	Sh	Ax	Sh	Ax	Sh	Ax	Sh	Ax
Flox	7	7	6	6	6	5(2)	6	6
ACKO	7	6	7	7	7	7	6(1)	7
PCKO	5	8	7	7	7	7	6	7
DCKO	5	6	6	6	6	6	6	6

CCL7	Sh	Ax	Sh	Ax	Sh	Ax	Sh	Ax
Flox	7	7	6	6	6	7	6	6
ACKO	7	6	7	7	7	7	7	7
PCKO	5	8	7	7	6(1)	7	5(1)	7
DCKO	5	6	6	6	6	6	6	5(1)

CCL12	Sh	Ax	Sh	Ax	Sh	Ax	Sh	Ax
Flox	6(1)	7	6	6	6	7	6	6
ACKO	7	6	7	7	6(1)	7	7	7
PCKO	5	7(1)	7	7	7	7	6	7
DCKO	5	6	6	6	6	6	6	6

The number of outlier values excluded are in parentheses

## Results

### CCL2 CKOs suggest broad expression of CCL2

After breeding our three CKO strains, we first looked at *Ccl2* expression in the DRG and DN in response to a unilateral sciatic nerve injury to confirm that the CKO strains decreased *Ccl2* as expected. *Ccl2* expression increases within hours of sciatic nerve transection [12, 19, 32] and peaks 1–2 days post-injury (DPI) [19]. We used Taqman qPCR assays to measure the level of *Ccl2* expression at both 1 and 2 DPI in the three CKOs relative to Flox controls. In the DRG, as expected, ACKOs showed a significant decrease in expression in both injured (i.e., axotomized) and contralateral non-injured (i.e., sham surgery) tissue relative to controls (Fig. 2A, B). Somewhat surprisingly, PCKOs also showed a significant decrease in both injured and uninjured DRG *Ccl2* expression suggesting that there are Schwann cell-related cells within the DRG that also make a significant amount of *Ccl2* (Fig. 2A, B). We examined P0-dependent reporter expression in the DRG (data not shown) and saw what appeared to be satellite glial cells expressing the reporter. After an injury, these cells, as well as neurons, are likely making *Ccl2*, and they may account for the small (though not statistically significant) injury-induced increases in *Ccl2* in the ACKOs. Finally, DCKOs also showed a significant reduction in *Ccl2* in the injured and non-injured tissue, although there is still detectable *Ccl2*, which leaves the possibility that still other cells produce the chemokine. In the DN, surprisingly, none of the CKOs showed reductions of *Ccl2*, most notably the PCKOs (Fig. 2C, D). This suggests that Schwann cells make a minor contribution to *Ccl2* expression in the DN after injury.

**Fig. 4:**
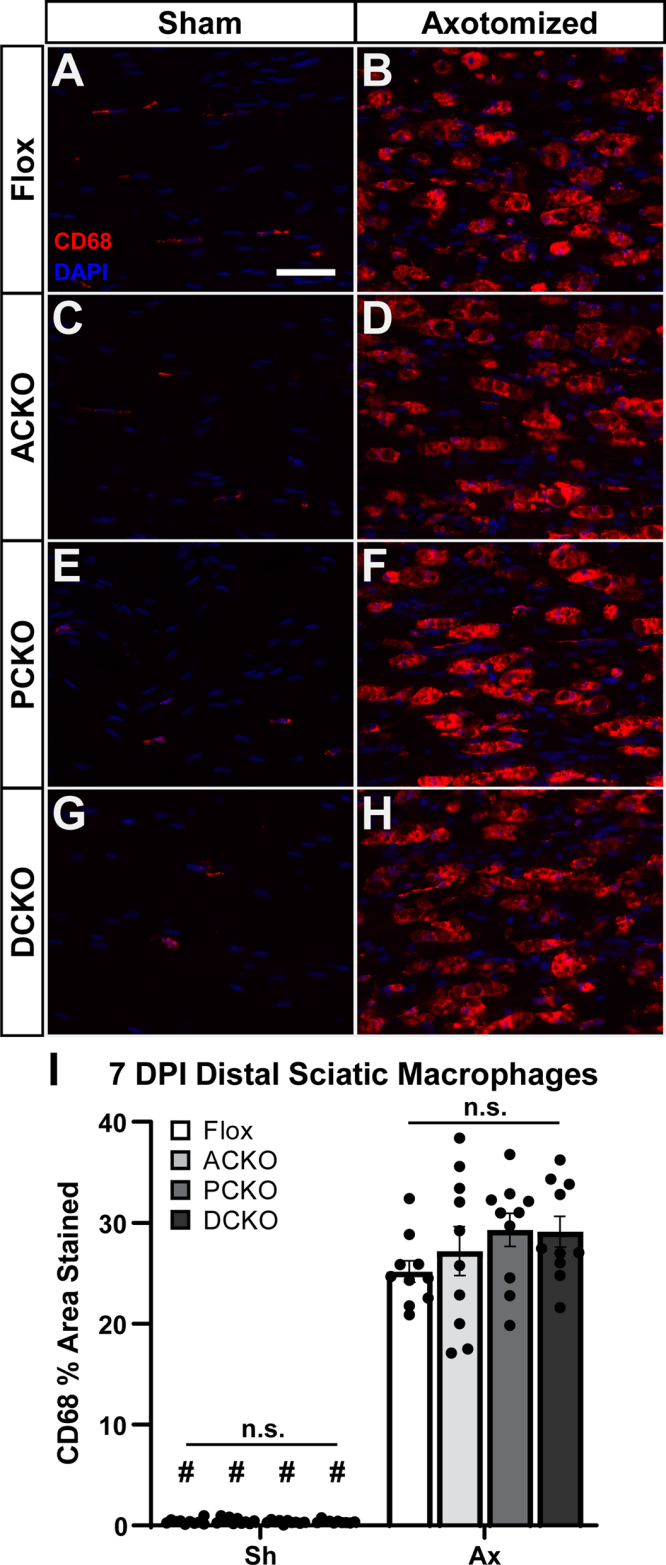
Distal sciatic macrophage accumulation and activation is unaffected in the nerve-targeted PCKO and DCKO 7 days after injury. **A**–**H** Representative images showing macrophages marked by CD68 immunostaining in the distal sciatic nerve ipsilateral (**B**, **D**, **F**, **H**) and contralateral (**A**, **C**, **E**, **G**) to a sciatic nerve transection. All genotypes have an increase in macrophages after injury (**A** vs. **B**; **C** vs. **D**; **E** vs. **F**; and **G** vs. **H**) and no differences in macrophages among genotypes either before (**A**, **C**, **E**, and **G**) or after (**B**, **D**, **F**, and **H**) injury. Scale bar is 50 μm. **I** Macrophages were quantified by CD68 area and plotted as mean ± SEM, *n* = 10 per group. # Indicates a significant (*p* < 0.05) difference between Sh and Ax within a genotype

At the same time, we examined macrophage recruitment to the DRG (Fig. 3) and DN (Fig. 4) after injury. Macrophage numbers reach their maximum between 3 and 7 DPI [33]; therefore, we examined recruitment at 7 DPI. Based on the paper by Kwon et al. [21], which shows a complete abolition of macrophage accumulation in *Ccl2* KOs 7 DPI, we expected to see reductions in DRG macrophages, since *Ccl2* is significantly reduced in all CKOs. However, there was no change in macrophage accumulation in L4 DRGs in any of the CKOs relative to Flox controls (Fig. 3I). We also looked at accumulation in the sciatic nerve, although since we did not observe a knockdown of *Ccl2* in our qPCR assays we did not expect to see a reduction in macrophages. Indeed, accumulation in the DN was unchanged in the CKOs (Fig. 4I).

### Macrophage function and peripheral axon regeneration are apparently normal in the CCL2 CKOs

**Fig. 5:**
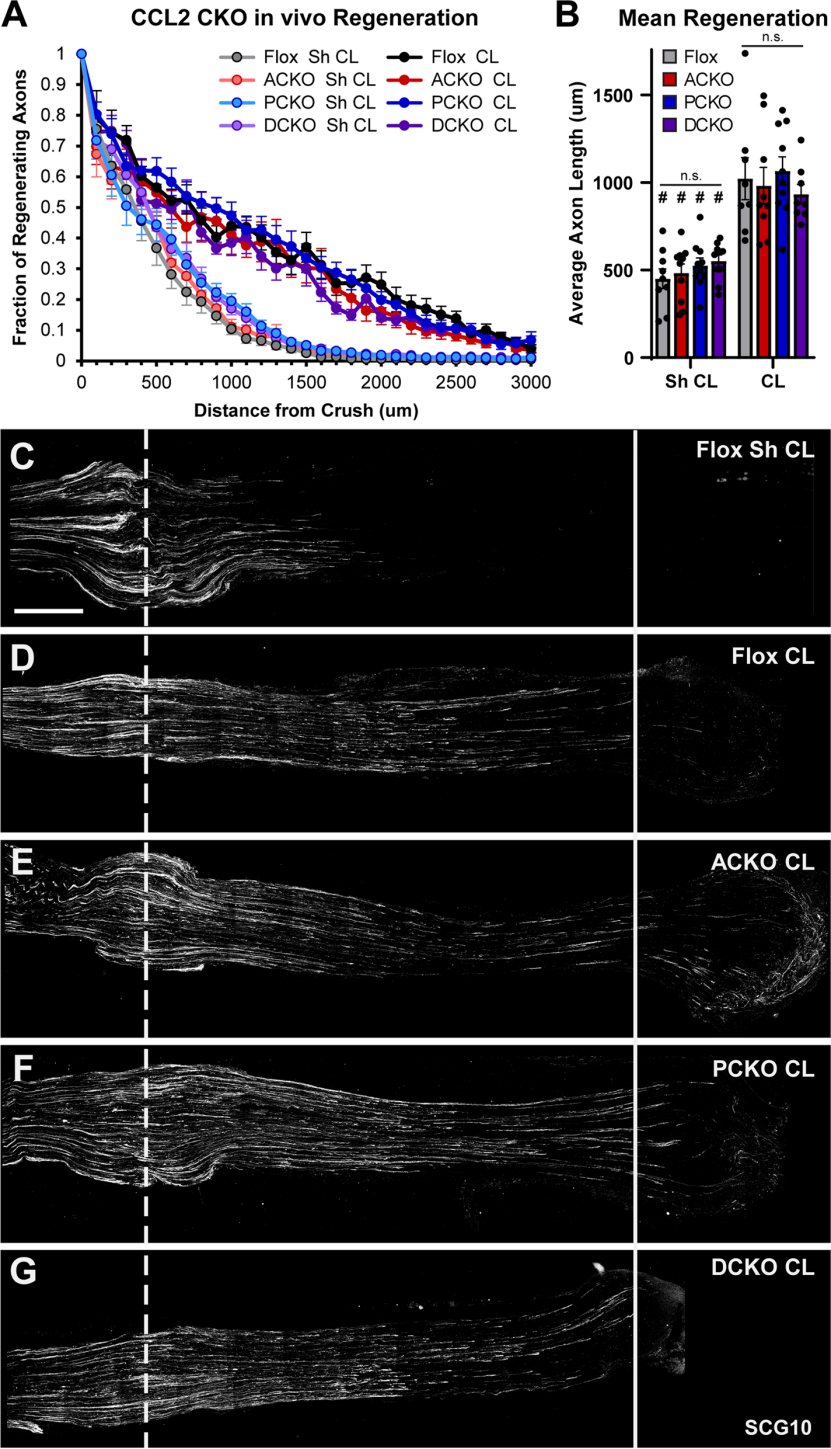
Conditioned and unconditioned in vivo regeneration is unaffected in all the conditional *Ccl2* knockouts. Conditioned nerves (CL) were transected 7 days prior to the test lesion crush and allowed to regenerate for 2 days. Unconditioned nerves (Sh CL) were given a sham surgery on the day of the conditioning lesion and were then crushed 7 days later and allowed to regenerate for 2 days. **A** Axon regeneration expressed as the fraction of axons relative to the crush site every 100 μm. Each point is the mean fraction ± SEM. **B** Mean regeneration determined by integrating regenerating axon fluorescence to find the average axon length for each nerve. # Indicates a significant (*p* < 0.05) difference between the Sh CL (unconditioned) and CL (conditioned) regeneration within a genotype. There were no significant differences between genotypes within an injury condition. For **A** and **B**, the analysis ends at 3000 μm from the crush because that is the length of the shortest nerve segment. For **A** and **B**, the n are: Flox Sh = 9, Flox CL = 8, ACKO Sh = 10, ACKO CL = 9, PCKO Sh = 10, PCKO CL = 10, DCKO Sh = 10, DCKO CL = 8. One CL nerve was excluded from analysis of each of the Flox CL, ACKO CL, and DCKO CL groups for violating assumption 5 (see “Materials and methods”). **C**–**G** Representative images of regenerating nerves stained with SCG10. Unconditioned growth (**C**), representing neuron intrinsic regeneration rate, was the same amongst all genotypes. Conditioned growth (**D**–**G**) was also the same across genotypes and increased compared to unconditioned controls (compare to **C**). The dotted line indicates the center of the crush site which was considered to be 500 μm wide, and the solid line is 3000 μm from the crush. Scale bar is 500 μm

**Fig. 6:**
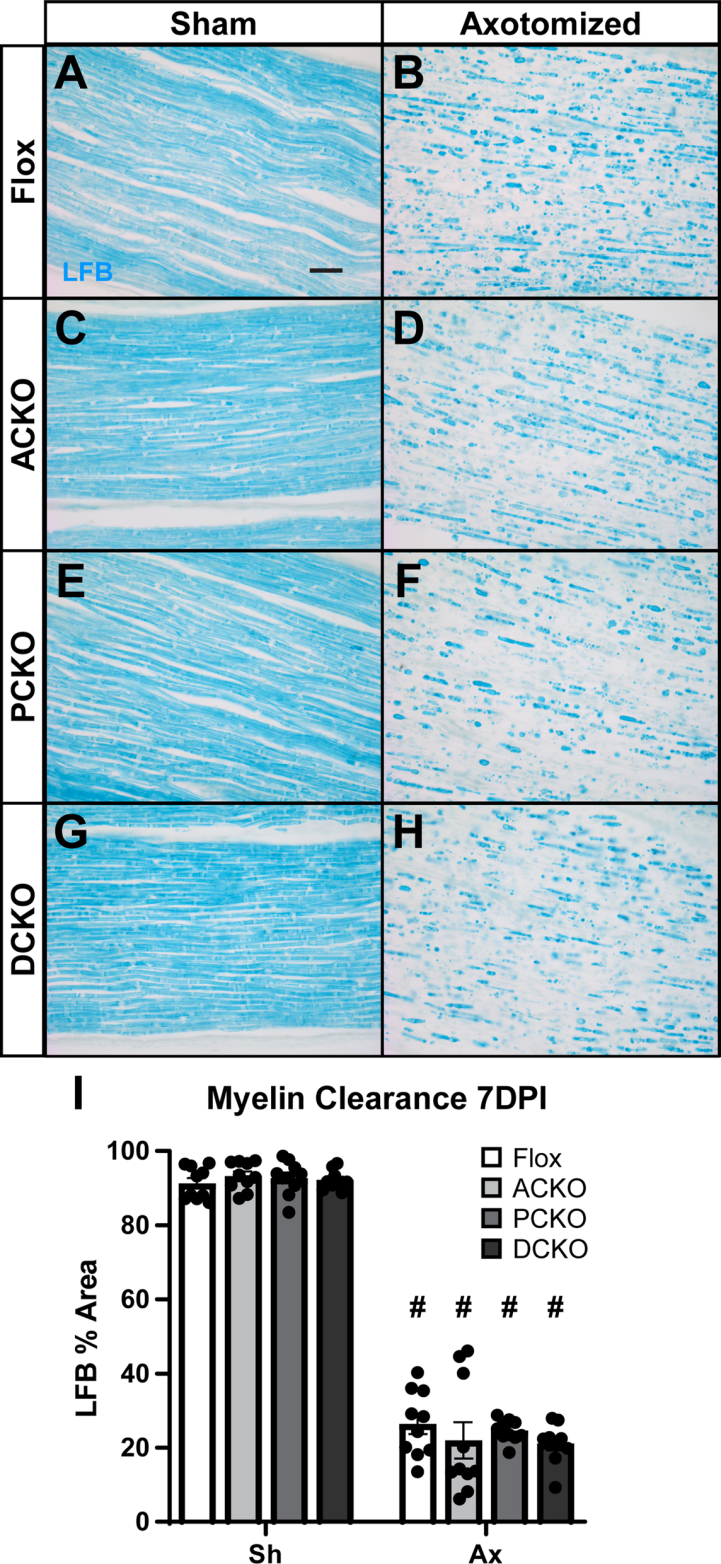
Myelin clearance in the distal sciatic nerve is unaffected in all the conditional *Ccl2* knockouts 7 days after injury. **A**–**H** Representative images of myelin stained with Luxol Fast Blue (LFB) in uninjured (**A**, **C**, **E**, **G**) and injured (**B**, **D**, **F**, **H**) sciatic nerves. In all genotypes, myelin has almost completely degenerated and been cleared by 7 DPI. Scale bar is 50 µm. **I** Myelin clearance was quantified by LFB area and plotted as mean ± SEM. # Indicates a significant (*p* < 0.05) difference between Sh and Ax within a genotype. *n* = 10 for all genotypes except DCKO *n* = 9

Although macrophage accumulation was normal in all CKOs, it was possible that these immune cells were not functionally normal. Kwon et al. [21] showed, using function blocking antibodies, that CCL2 signaling is required for macrophages to enter a growth-promoting state, which stimulates CL-enhanced growth. Given the large reduction in *Ccl2* in the CKO DRGs, the macrophages may be present but not able to promote growth. To that end, we tested if peripheral axon regeneration and CL-enhanced growth were altered in vivo (Fig. 5). In these assays, we transected the right sciatic nerve distally (the CL) and waited 7 days, at which point we crushed both sciatic nerves proximally (the test lesion) and allowed 2 days for regeneration (Fig. 1A). Using this paradigm, we can measure both unconditioned and conditioned peripheral axon regeneration in the Sham and CL groups, respectively. When we measured regeneration in the CKOs, we found that neither conditioned nor unconditioned regeneration is altered (Fig. 5A, B). We also examined myelin clearance in the distal nerves, another process assisted by macrophages, and found that it is also unaltered in the CKOs (Fig. 6). From these results, it seems that the remaining CCL2 or other macrophage chemokines are sufficient to promote macrophage accumulation and the CL response. This CCL2 must be coming from as yet unidentified cells in both the DRG and DN. We next sought to identify its additional sources with the hope that we could create complete site-specific KOs in the DRG and DN.

### CCL2 is expressed in many cell types in DRGs and the DN

Our lab has tried unsuccessfully over several years to visualize and localize CCL2 in DRGs and DNs with several different antibodies. In previous mouse studies, in situ hybridization was used to detect CCL2 in the DRG and DN without counterstains to definitively prove that the detected CCL2 was restricted to a particular cell type(s) [24, 34]. Fortunately, there is a new transgene encoding CCL2 fused to a viral 2A cleavage sequence and the fluorophore mCherry [29]. The 2A sequence causes the mCherry to be “cleaved” co-translationally from CCL2 and thus marks CCL2 expressing cells with mCherry. This allowed us to detect sources of CCL2 without a CCL2 antibody, and furthermore, the signal could be amplified using antibodies that bind mCherry. In addition, the entire CCL2–2A-RFP coding sequence is flanked by loxP sites which allowed us to detect the remaining sources of CCL2 in the presence of both Cre drivers (i.e., the DCKOs). Using these CCL2–RFP and CCL2–RFP DCKO mice, we labeled CCL2-expressing cells with one of two antibodies against RFP (see “Materials and methods”) and co-labeled with various cell type specific markers. The half-life of fluorescent proteins is about 1 day, and since *Ccl2* mRNA expression peaks 1–2 DPI, we looked for RFP labeled cells 24 h later at 2 and 3 DPI.

**Fig. 7:**
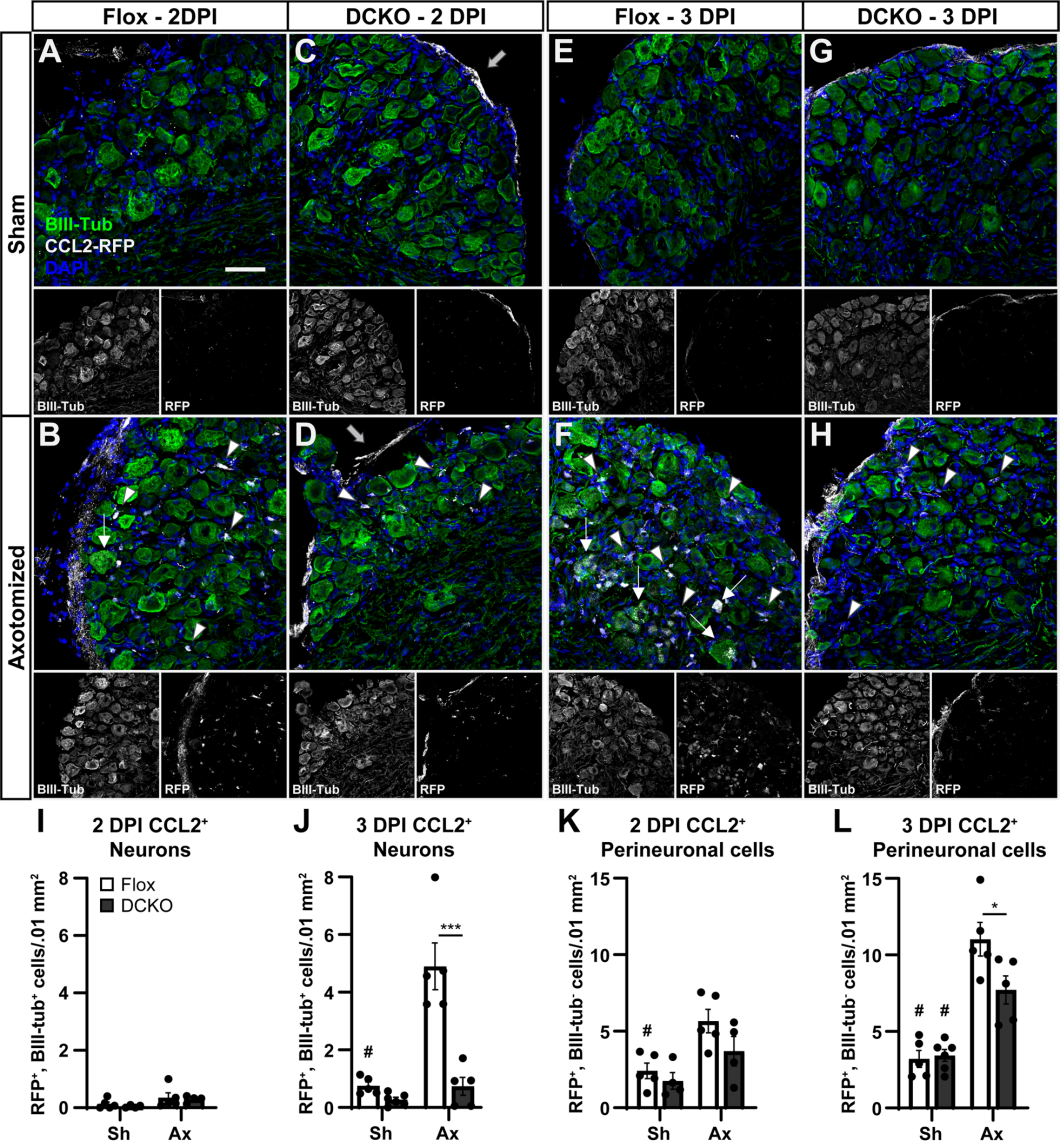
CCL2 is expressed in both neurons and perineuronal cells in the DRG after injury. **A**–**H** Representative images of uninjured (**A**, **C**, **E**, **G**) and injured (**B**, **D**, **F**, **H**) DRGs stained for the co-translated RFP to localize CCL2 expression at 2 (**B**, **D**) and 3 (**F**, **H**) DPI. Very little CCL2 is present at baseline (**A**, **C**, **E**, **G**). CCL2 is rapidly upregulated in various perineuronal cells (arrowheads) in both the Flox control (**B**, **F**) and DCKO (**D**, **H**). Expression in neurons lags behind perineuronal expression in Flox controls (arrows, **B**, **F**), and little to no neuronal expression is seen in DCKOs (**D**, **H**). Large gray arrows indicate the likely meningeal expression. Scale bar is 50 μm. **I**–**L** Quantification of cells making CCL2 at 2 (**I**, **K**) and 3 (**J**, **L**) DPI in the cell body area of the DRGs. There is an apparent delayed onset of CCL2 expression in neurons (Flox Ax, **I** vs. **J**) when compared to perineuronal cells (**K**, **L**). The DCKOs show a strong CCL2 KO in neurons (**I**, **J**) and a trend toward fewer CCL2 expressing perineuronal cells (**K**, **L**). Individual images for the BIII-Tubulin and RFP channels are also displayed below each merged image (**A**–**H**). Data are the mean ± SEM, *n* = 5 for all groups except DCKO 2 DPI, where *n* = 4. One pair of 3 DPI Flox and one pair of 3 DPI DCKOs stained too badly to quantify and were excluded, and one 2 DPI DCKO Ax DRG was lost in sectioning. # Indicates a significant (*p* < 0.05) difference between Sh and Ax within a genotype and * indicates a significant difference between indicated genotypes within an injury condition (*p < 0.05, *** *p* < 0.001)

In the DRG, we identified neurons by co-labeling with βIII-tubulin, DAPI, and RFP. We also identified CCL2 expressing non-neuronal cells, which were nuclei associated with an extra-neuronal RFP signal (Fig. 7). The number of non-neuronal RFP^+^ cells was increased relative to sham at both 2 and 3 DPI (Fig. 7K, L), while the number of RFP^+^ neurons did not increase until 3 DPI in the Flox controls (Fig. 7I, J). This implies that non-neuronal cells such as macrophages, satellite glial cells, and other stromal cells are the first producers of CCL2. There was also a small decrease in the number of RFP^+^ non-neuronal cells (Fig. 7K, L) and a qualitative decrease in RFP signal intensity (Fig. 7: arrowheads in B vs. D and F vs. H) in the DCKOs relative to Flox controls. When considered with our observations that P0-Cre expresses in satellite glial cells and that PCKOs have decreased DRG CCL2 expression (Fig. 2), it suggests that the RFP decrease in non-neuronal cells in DCKOs is due to a loss of expression in satellite glial cells. It also implies that there are at least three cell types that express CCL2 in the DRG. The remaining cells in the DCKOs are most likely macrophages and possibly stromal cells, such as fibroblasts or endothelia [16, 35]. Finally, we often observed RFP signal in a thin membrane surrounding the DRG (e.g., Fig. 7C, D gray arrows), which is likely a layer of the meninges also making CCL2. The diverse cells expressing CCL2 in the DRG makes creating a site-specific knockout by targeting particular cell types extremely difficult.

**Fig. 8:**
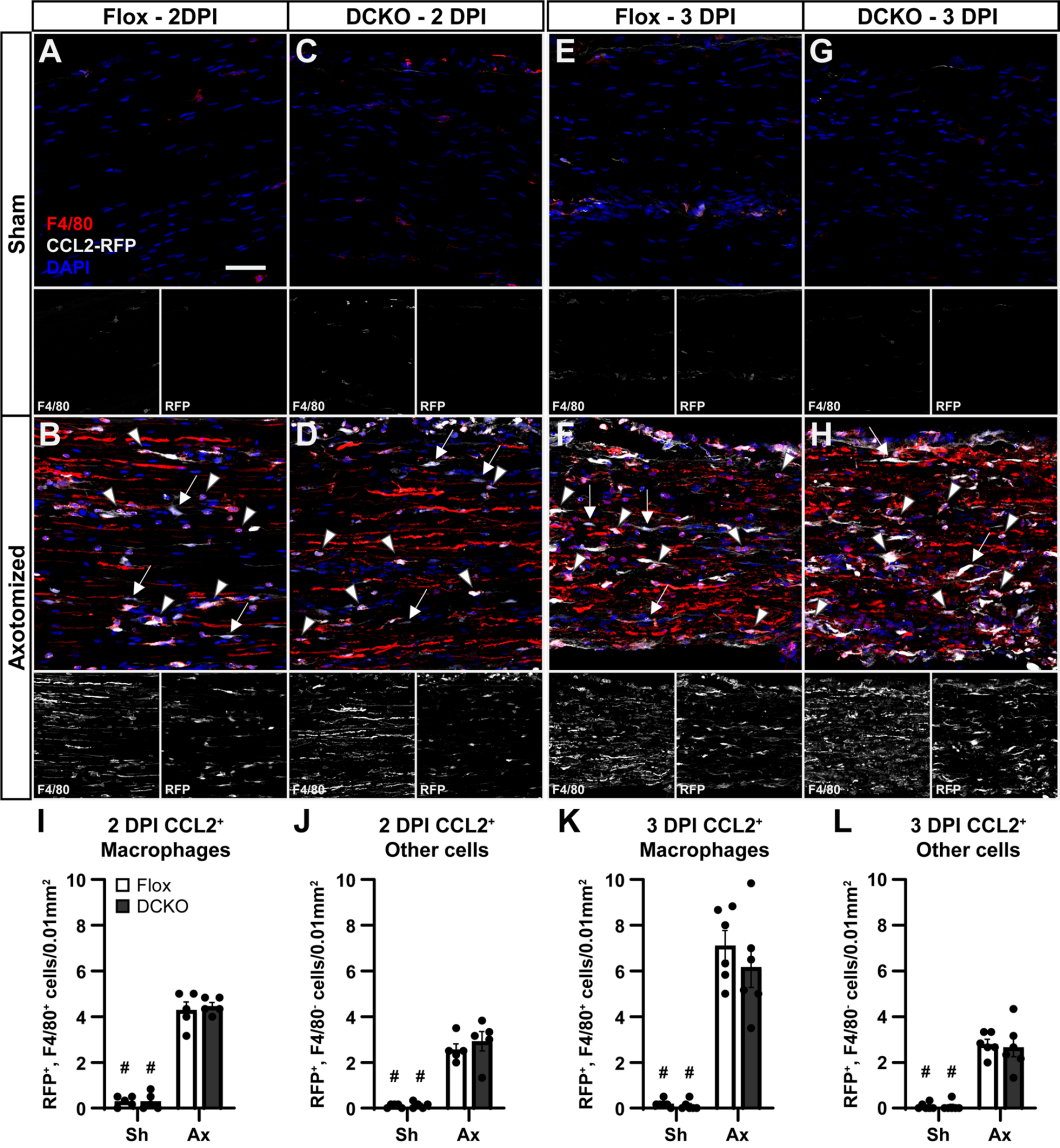
CCL2 is expressed primarily in macrophages in the distal sciatic nerve after injury. **A**–**H** Representative images of uninjured (**A**, **C**, **E**, **G**) and injured (**B**, **D**, **F**, **H**) distal nerves stained for the co-translated RFP to localize CCL2 expression at 2 (**B**, **D**) and 3 (**F**, **H**) DPI. Very little CCL2 is present at baseline (**A**, **C**, **E**, **G**). Many F4/80^+^ cells express CCL2 at both 2 (**B**, **D**; arrowheads) and 3 (**F**, **H**; arrowheads) DPI. Other cells that are likely Schwann cells and other stromal cells (arrows; **B**, **D**, **F**, **H**) also express CCL2 after injury. **I**–**L** Quantification of cells making CCL2 at 2 (**I**, **J**) and 3 (**K**, **L**) DPI. The majority of CCL2^+^ cells are F4/80^+^ macrophages at both 2 (**I** vs. **J**) and 3 (**K** vs. **L**) DPI, and CCL2^+^ macrophages increase over the course of the injury response (**I** vs. **K**). The lack of a decrease in non-macrophage CCL2^+^ cells in the DCKOs compared to controls (**J**, **L**) suggests Schwann cells make little contribution to CCL2 expression. Individual images for the F4/80 and RFP channels are also displayed below each merged image (**A**–**H**). Data are the mean ± SEM, *n* = 5 for the 2 DPI groups and *n* = 6 for the 3 DPI groups. # Indicates a significant (*p* < 0.05) difference between Sh and Ax within a genotype

In the sciatic nerve, we identified CCL2 expressing macrophages with F4/80, DAPI, and RFP. We attempted to label Schwann cells with a Sox10 antibody as well but the signal to noise ratio was never high enough to allow for confident quantification. Thus, we quantified macrophages and non-macrophages expressing CCL2–RFP (Fig. 8). Surprisingly, macrophages were the majority of CCL2 expressing cells at both 2 (Fig. 8I vs. J) and 3 (Fig. 8K vs. L) DPI. In both Flox controls and DCKOs, macrophages were two-thirds to three-fourths of the CCL2–RFP^+^ cells. Furthermore, there was no decrease in CCL2–RFP^+^ non-macrophages in the DCKOs which suggests that Schwann cells make only a minor contribution to *Ccl2* expression, if any, in the injured DN. This supports our finding that neither PCKOs nor DCKOs have decreased *Ccl2* expression in the sciatic nerve after injury (Fig. 2C, D). It is likely that the other CCL2–RFP^+^ cells are again stromal cells, such as fibroblasts and endothelia.

### Macrophage function and peripheral axon regeneration are normal in the absence of CCL2

Our finding that macrophages are a significant source of CCL2 in both the DRG and DN also complicated our original goal of inhibiting macrophage recruitment to specific sites by removing tissue specific CCL2 sources. Furthermore, our finding that macrophage recruitment in the DRG is unchanged in all CKOs was confounding. We were perplexed by the normal recruitment to DCKO DRGs (Fig. 3G–I), where the amount of *Ccl2* mRNA after injury is less than in the uninjured Flox control DRGs (Fig. 2A, B), which contain relatively few macrophages (Fig. 3A). On the other hand, the result in Kwon et al., [21] was clear cut: there was not the slightest increase in macrophage numbers in *Ccl2* KO DRGs relative to uninjured controls. It was difficult for us to reconcile their finding that CCL2 was essential for macrophage recruitment with our finding that recruitment was unperturbed in a nearly complete DRG knockdown. Therefore, we decided to re-examine macrophage recruitment in global *Ccl2* KOs to confirm that knocking out *Ccl2* was a viable strategy to prevent macrophage recruitment.

**Fig. 9:**
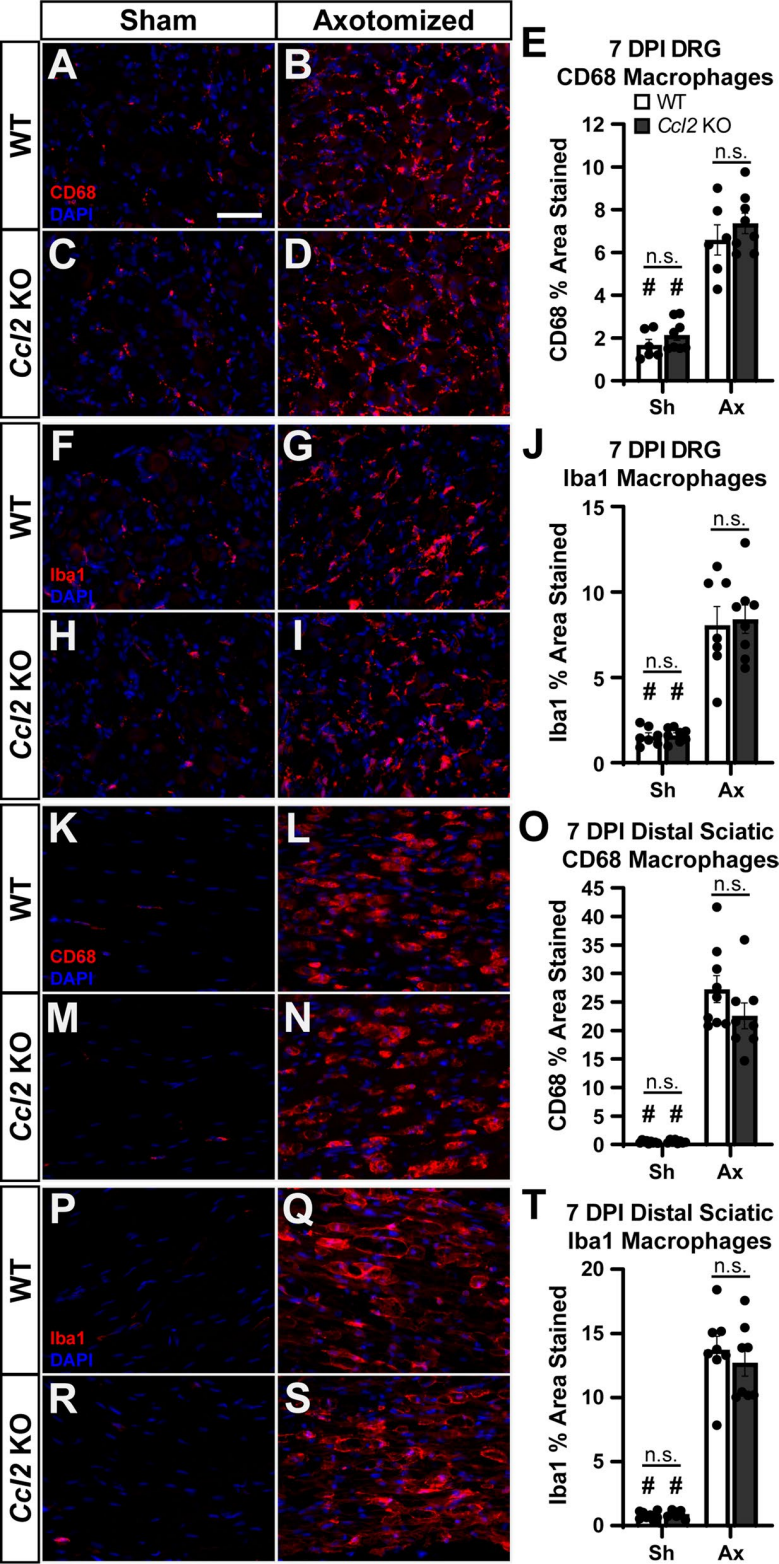
CCL2 is not required for macrophage recruitment either to the DRG or distal sciatic nerve 7 DPI. **A**–**D** Representative images showing macrophages marked by CD68 immunostaining in L4 DRGs ipsilateral (**B**, **D**) and contralateral (**A**, **C**) to a sciatic nerve transection. *Ccl2* KOs show an increase in macrophages after injury comparable to WT controls (**B** vs. **D**). **E** Macrophages were quantified by CD68 area and plotted as mean ± SEM. There are no differences between genotypes before or after injury. WT *n* = 6, *Ccl2* KO *n* = 8. **F**–**I** Representative images showing macrophages marked by Iba1 immunostaining in L4 DRGs ipsilateral (**G**, **I**) and contralateral (**F**, **H**) to a sciatic nerve transection. Again, *Ccl2* KOs show an increase in macrophages after injury comparable to WT controls (**G** vs. **I**). **J** Macrophages were quantified by Iba1 area and plotted as mean ± SEM. There are no differences between genotypes before or after injury. WT *n* = 7, *Ccl2* KO *n* = 8. **K**–**N** Representative images showing macrophages marked by CD68 immunostaining in distal sciatic nerves ipsilateral (**L**, **N**) and contralateral (**K**, **M**) to a sciatic nerve transection. *Ccl2* KOs show an increase in macrophages after injury comparable to WT controls (**L** vs. **N**). **O** Macrophages were quantified by CD68 area and plotted as mean ± SEM. There are no differences between genotypes before or after injury. WT *n* = 9, *Ccl2* KO *n* = 8. **P**–**S** Representative images showing macrophages marked by Iba1 immunostaining in distal sciatic nerves ipsilateral (**Q**, **S**) and contralateral (**P**, **R**) to a sciatic nerve transection. Again, *Ccl2* KOs show an increase in macrophages after injury comparable to WT controls (**Q** vs. **S**). **T** Macrophages were quantified by Iba1 area and plotted as mean ± SEM. There are no differences between genotypes before or after injury. WT *n* = 8, *Ccl2* KO *n* = 8. # Indicates a significant (*p* < 0.05) difference between Sh and Ax within a genotype. Scale bar is 50 μm

To confirm the results from Kwon et al. [21], we replicated their methods as faithfully as possible. We acquired the same *Ccl2* KO strain from Jackson Laboratories, we performed a sciatic nerve transection as they did, and we used two different antibodies to label macrophages: an anti-CD68 used regularly by our laboratory and the anti-Iba1 clone from the same company used in their paper. We looked at macrophage recruitment 7 DPI in both L4 DRGs and the DN and found no deficit in macrophage recruitment in either tissue using either antibody in the *Ccl2* KOs (Fig. 9). This result extends our CKO findings and suggests that CCL2 is not required for macrophage recruitment after nerve injury.

**Fig. 10:**
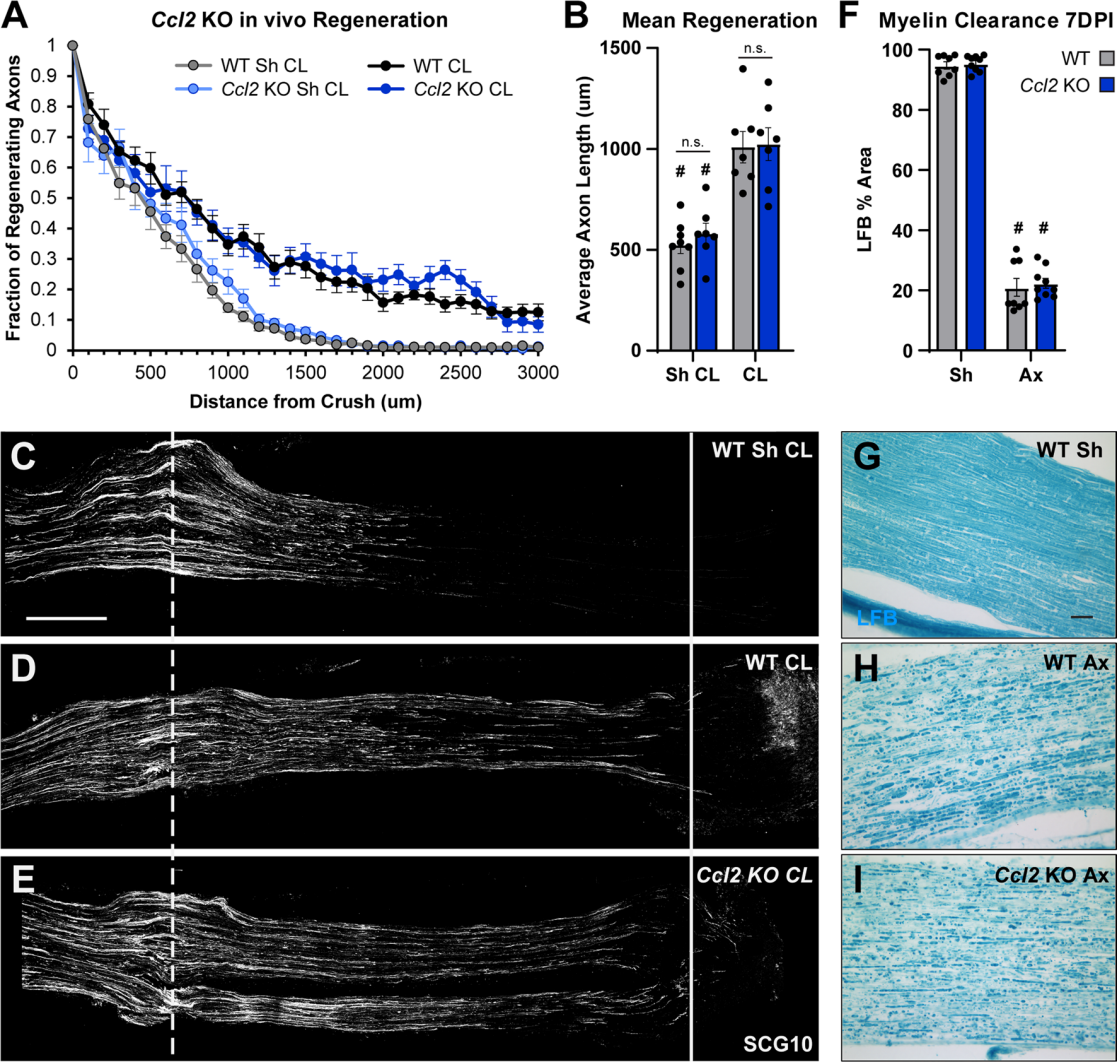
Degeneration and regeneration are functionally normal in the absence of CCL2. Conditioning and in vivo regeneration were done as in Fig. 4. **A** Axon regeneration expressed as the fraction of axons relative to the crush site every 100 μm. Each point is the mean fraction ± SEM. **B** Mean regeneration determined by integrating regenerating axon fluorescence to find the average axon length for each nerve. Average axon lengths are plotted as mean ± SEM. For **A** and **B**, the analysis ends at 3000 μm from the crush, because that is the length of the shortest nerve segment. *n* = 7 nerves per injury condition per genotype, except WT Sh CL *n* = 8. One WT CL nerve was excluded from analysis for violating assumption 5 (see “Materials and methods”). **C**–**E** Representative images of regenerating nerves stained for SCG10. Unconditioned growth (**C**), representing neuron intrinsic regeneration rate, was the same in both genotypes. Conditioned growth (**D**, **E**) was also the same in *Ccl2* KOs as in WT and increased compared to unconditioned controls (compare to **C**). The dotted line indicates the center of the crush site which was considered to be 500 μm wide, and the solid line is 3000 μm from the crush. Scale bar is 500 μm. **F** Myelin clearance, quantified by LFB area and plotted as mean ± SEM. WT *n* = 8, *Ccl2* KO *n* = 9. **G**–**I** Representative images of myelin stained with LFB in uninjured (**G**) and injured (**H**, **I**) sciatic nerves. In both genotypes, myelin has almost completely degenerated and been cleared by 7 DPI. # Indicates a significant (*p* < 0.05) difference between the Sh CL (unconditioned) and CL (conditioned) regeneration within a genotype

As described above, Kwon et al. also suggested that CCL2 was necessary for stimulating a growth promoting macrophage phenotype and thus CL-enhanced regeneration. Therefore, despite the fact that we observed normal macrophage accumulation, the possibility remained that the macrophages were dysfunctional. We tested this by examining in vivo regeneration and myelin clearance in *Ccl2* KOs. We found that 7 DPI myelin clearance is normal in *Ccl2* KOs (Fig. 10F–I), and more importantly, we found that in vivo peripheral regeneration and CL-enhanced regeneration are both normal in these KO mice (Fig. 10A–E). Therefore, our data indicate that CCL2 is not necessary for inducing the CL response in vivo.

### Two other CCR2 chemokines are expressed in the DRG and DN after injury

**Fig. 11:**
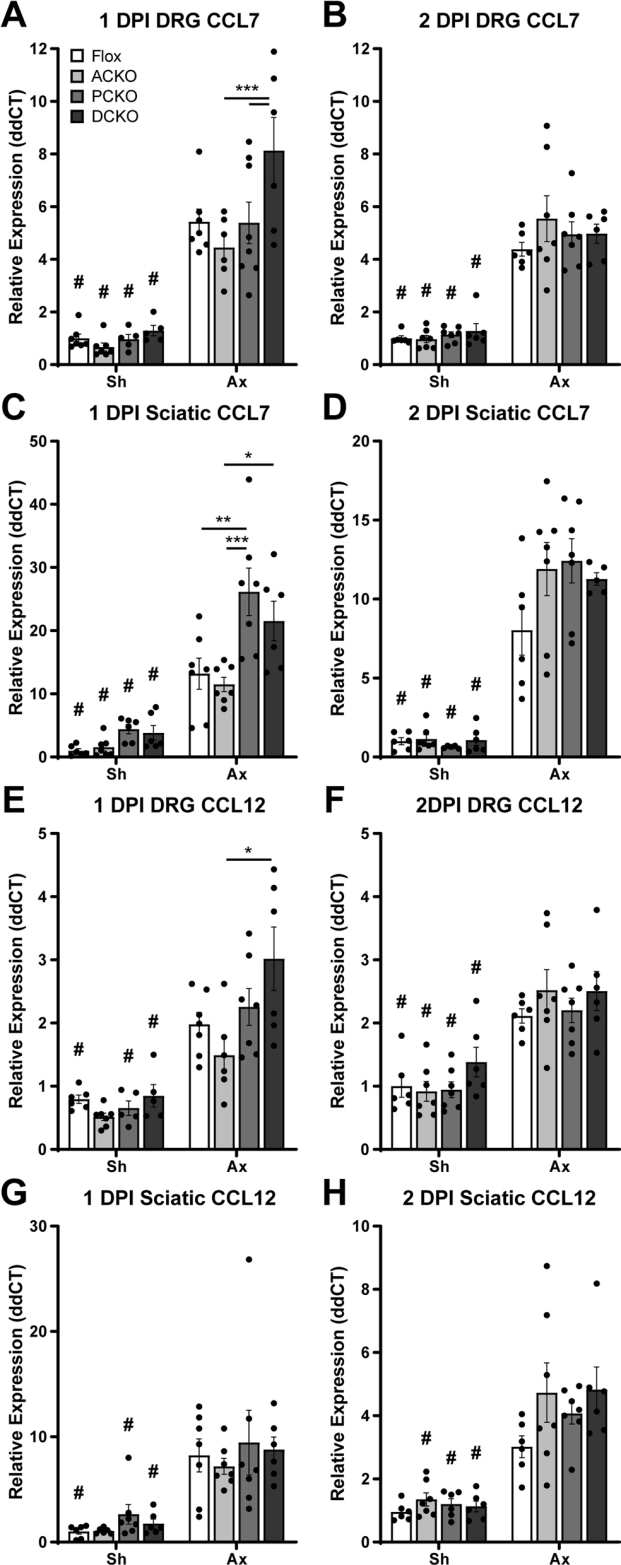
Other CCR2 chemokines are upregulated in response to injury in both the DRG and sciatic nerve. **A**–**D** *Ccl*7 mRNA is upregulated within 1 day after injury in both the DRG (**A**, **B**) and distal sciatic nerve (**C**, **D**). **E**–**H** *Ccl12* mRNA is also upregulated within 1 day after injury in both the DRG (**E**, **F**) and distal sciatic nerve (**G**, **H**). # Indicates a significant (*p* < 0.05) difference between Sh and Axwithin a genotype and * indicates a significant difference between indicated genotypes within an injury condition (*p < 0.05, **p < 0.01, ***p < 0.001)

Finally, since we find that CCL2 is not necessary for macrophage recruitment, we hypothesized that other factors compensate for the loss of CCL2. The macrophage chemokine receptor CCR2 seems to be required for recruitment of circulating monocytes after nerve injury [12, 13, 36], so we looked for other chemokines that signal through this receptor. CCL7, CCL8, and CCL12 are three chemokines that might compensate for loss of CCL2 as they all have the ability to bind CCR2 and stimulate chemotaxis in vitro [18, 37–39]. Indeed, there are a few reports that have detected expression of these chemokines in the DRG up to 1 day after nerve injury [40, 41], and our lab has previously reported that CCL8 is upregulated in the DRG after injury [11]. In addition, CCL2, CCL7, and CCL12 have been found in areas of the brain after traumatic brain injury [42]. Thus, we used qPCR to measure the expression of *Ccl7* (also known as MCP-3) and *Ccl12* (also known as MCP-5) in the DRG and DN 1 and 2 DPI. We found that both chemokines are upregulated in response to injury in both tissues (Fig. 11). This confirms and extends what others have observed and could explain why macrophages are still recruited and activated in the absence of CCL2.

### Resident macrophages and alternative recruitment mechanisms compensate for loss of CCR2 signaling

These findings suggest three possible mechanisms for the unaltered inflammatory response we observe in the *Ccl2* KOs: one, a combination of other CCR2 chemokines, specifically CCL7, CCL8, and CCL12, compensate for CCL2 and are able to recruit WT numbers of monocytes from circulation; two, recruited macrophages require CCL2 signaling but residents are able to proliferate and compensate for the loss of CCL2 and recruited macrophages; and three, both compensatory chemokines and resident macrophage proliferation lead to normal macrophage accumulation in the absence of CCL2. To further investigate the role of CCR2 ligands in DRG and DN inflammation after PNI, we used a new *Ccr2*^gfp^ knock-in/knockout strain, which has an eGFP allele knocked into the endogenous CCR2 locus. In heterozygotes (CCR2^gfp^ het), CCR2 expressing cells will be marked by GFP and function normally. However, in homozygotes (CCR2^gfp^ KO) CCR2 expressing cells will still be marked by GFP but will not have CCR2 function [31]. In theory the GFP signal will distinguish recruited macrophages (derived from circulating Ly6C^hi^, CCR2^+^ monocytes [43, 44]) from resident macrophages, which are CCR2^−^ [45], in WT and CCR2 KO backgrounds. It should be noted that there is another subset of circulating “nonclassical” monocytes which are Ly6C^lo^, CX3CR1^hi^, and CCR2^lo^ or CCR2^−^. These hematogenous monocytes may lack a GFP signal and could be mistaken for resident derived cells if they were recruited after injury. However, this monocytic subtype responds poorly or not at all to inflamed locations, and thus the vast majority of GFP^−^ macrophages will be resident derived [43, 44]. This will allow us to distinguish between the three possible compensatory mechanisms.

One possible confound is that resident macrophages in both the DRG and sciatic nerve can be replenished by CCR2^+^ circulating monocytes and thus may express GFP, while they differentiate into CCR2^−^ tissue resident macrophages [44, 46, 47]. To control for this, we quantified GFP^+^, CD68^+^ cells as a percentage of the CD68 population in both uninjured (i.e., sham) and injured tissue. Double positive residents should not proliferate faster or slower than CD68 only residents and thus the percentage should remain the same (or decrease if GFP is diluted by cell divisions) if no new CCR2^+^ monocytes are recruited. Importantly, an increase in the percentage is a definite indication that circulating monocytes are being recruited. Thus, we used CCR2^gfp^ het and KO animals to examine how CCR2 signaling affects the recruited macrophage response 7 DPI.

**Fig. 12:**
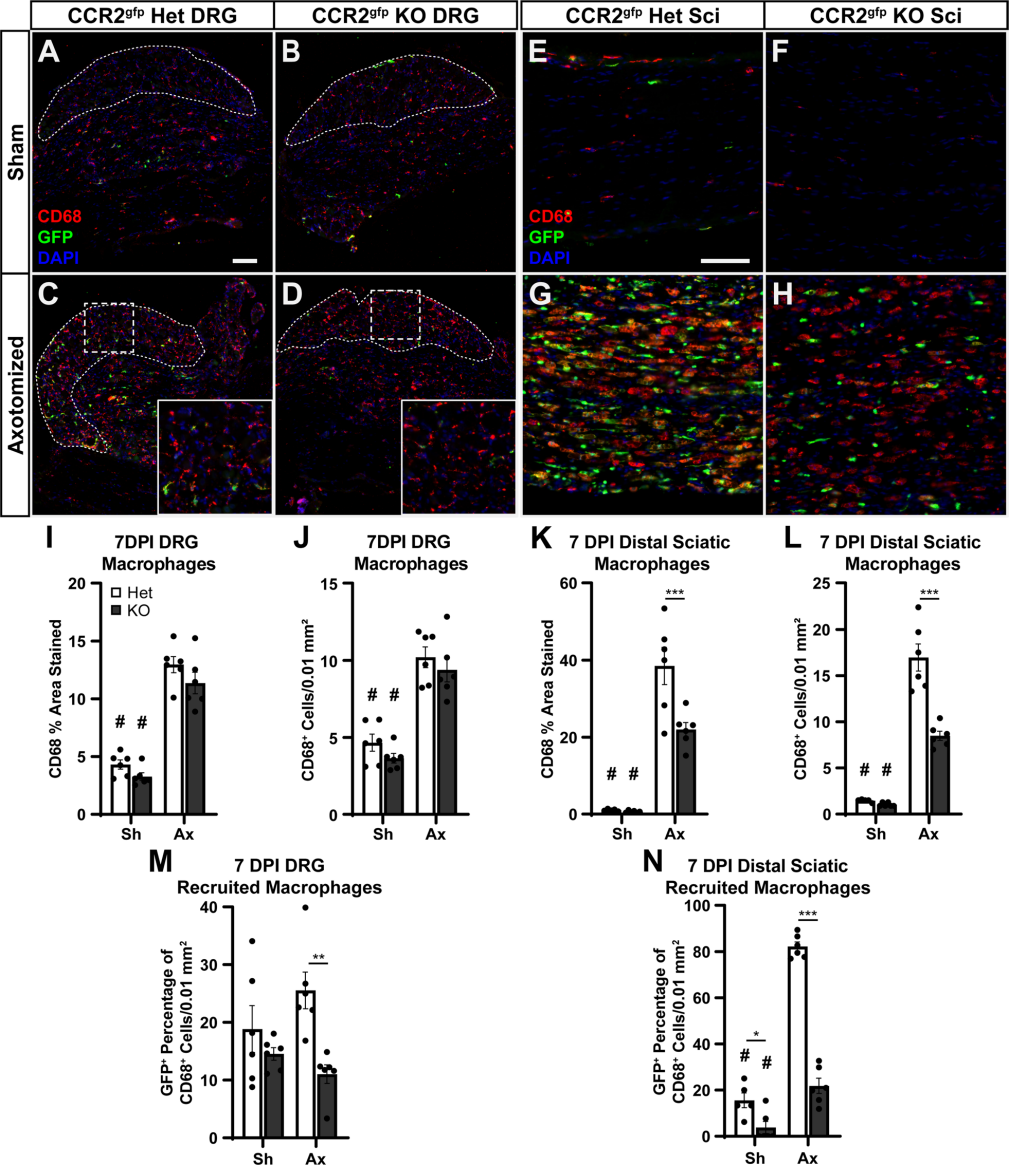
Resident macrophages and alternative signaling mechanisms compensate for the loss of CCR2. **A**–**D** Representative images of CCR2^gfp^ het and CCR2^gfp^ KO DRGs contralateral (**A**, **B**) and ipsilateral (**C**, **D**) to a sciatic nerve transection. All macrophages are marked by CD68 immunostaining and monocyte derived macrophages are marked by GFP immunostaining. Injured DRGs in both genotypes show an increase in CD68 macrophages compared to the contralateral control (**C**, **D** vs. **A**, **B**). The CCR2^gfp^ het DRGs have more GFP^+^ cells and show a small increase after injury (**C**). Dotted tracings indicate the cell body area and are representative of the area quantified. Dotted squares indicate the location of the insets and are 200 by 200 µm. Scale bar is 100 µm. **E**–**H** Representative images of CCR2^gfp^ het and CCR2^gfp^ KO DNs contralateral (**E**, **F**) and ipsilateral (**G**, **H**) to a sciatic nerve transection. All macrophages are marked by CD68 immunostaining and recruited macrophages are marked by GFP immunostaining. Injured DNs in both genotypes show an increase in CD68 macrophages compared to their contralateral control (**G**, **H** vs. **E**, **F**) but macrophages are significantly reduced in CCR2^gfp^ KO DNs (**G** vs. **H**). The CCR2^gfp^ het DNs have more GFP^+^ cells and show a substantial increase after injury (**G**), while the CCR2^gfp^ KO DNs have a relative increase in resident CD68 only macrophages as well as a smaller increase in the double positive recruited macrophages (**H**). Scale bar is 100 µm. **I**–**L** Quantification of CD68 macrophages in the DRG cell body area (**I**, **J**) or the DN (**K**, **L**) by the percent area positively stained (**I**, **K**) and by cell counts (**J**, **L**). **M**, **N** Quantification of the percentage of recruited macrophages in the DRG cell body area (**M**) and in the DN (**N**). Percentages were calculated as the number of double positive cells over the CD68 positive cells. # Indicates a significant (*p* < 0.05) difference between Sh and Ax within a genotype and * indicates a significant difference between indicated genotypes within an injury condition (*p < 0.05, **p < 0.01, ***p < 0.001). All data are the mean ± SEM and *n* = 6 per injury condition per genotype

In the sciatic nerve, we observe 50% fewer macrophages in the injured CCR2^gfp^ KOs compared to the hets both by CD68 percent area stained and by CD68 cell counts (Fig. 12E–H, K, L) although there is still a significant increase relative to sham controls in both genotypes. We also observe that 70–80% of macrophages in injured het DNs are recruited from circulating monocytes (Fig. 12N). These findings are in line with previous reports [11, 36]. Interestingly, there is a significant increase in recruited macrophages in the injured KOs as well as a relative expansion of GFP^−^ resident macrophages. Yet, these mechanisms are unable to recruit and activate macrophages to the level of the het controls. These findings show that residents are likely able to increase their proliferation to compensate for deficits in recruited macrophages up to a certain limit, and that there are CCR2 independent mechanisms to recruit macrophages to the DN after injury. Although, some of the increase in GFP^−^ macrophages could be due to recruitment of nonclassical Ly6C^lo^ circulating monocytes. Furthermore, in the context of the unaltered macrophage response in CCL2 KOs, both CCR2 and non-CCR2 chemokines are likely compensating for the loss of CCL2 along with increased resident macrophage proliferation. In addition, since there are still recruited macrophages in the CCR2^gfp^ KO DNs, loss of any single CCR2 ligand cannot abolish macrophage recruitment in the nerve. This confirms that CCL2 is not required for the DN injury response.

In the DRG, we observed no significant change in the macrophage injury response in the CCR2^gfp^ KOs compared to het controls (Fig. 12A–D, I, J) within the cell body area of the DRG (outlined in Fig. 12A–D). This is surprising as it disagrees with previous reports that see a decrease in macrophages in CCR2 KO DRGs [11, 12]. However, we do observe a trend toward a ~ 10% decrease in macrophages in the CCR2^gfp^ KOs. Supporting the finding that the macrophage response is mostly unaffected in recruitment deficient CCR2^gfp^ KOs, as many as 95% of macrophages in the DRG after injury are resident derived (Fig. 12M). However, there is still an increase in GFP^+^ macrophages, though not significant, of ~ 10% in the injured hets compared to their sham control which suggests that while the majority of injury induced DRG macrophages are derived from residents, a small percentage may be derived from circulating monocytes. Indeed, a recent model using parabiosis to study resident vs. infiltrating macrophages in the DRG reported similar findings (Kalinski et al., [14]). The fact that most injury induced DRG macrophages are derived from residents and are activated in a *Ccr2* KO model, also implies that CCL2 is not necessary for macrophage recruitment or activation in the DRG.

## Discussion

This study has reexamined and expanded what is known about CCL2 in peripheral nerve injury and regeneration. CCL2 was thought to be essential for injury induced macrophage accumulation in vivo based on Fig. 4 from Kwon et al., [21]. From this finding, we hypothesized that macrophage accumulation could be prevented in either the DRG or DN by knocking out CCL2 from the cells producing it in each location. These cells were putatively neurons in the DRG and Schwann cells in the DN, so we created CKOs targeting neurons (ACKOs), Schwann cells (PCKOs), or both (DCKOs). Surprisingly, both ACKOs and PCKOs had decreased *Ccl2* mRNA in the DRG, suggesting that both neurons and satellite glia produce CCL2. We confirmed this finding with immunofluorescent staining in CCL2–RFP animals, which revealed RFP in both neurons and perineuronal cells. In DCKOs, neuronal staining was lost and perineuronal staining was decreased, supporting the interpretation that both neurons and glia produce CCL2. Furthermore, the residual *Ccl2* mRNA and RFP seen in the DCKOs is from another perineuronal cell type [16, 34] which are likely macrophages, since macrophages are a major source of CCL2 [35], and the remaining CCL2–RFP signal in DCKO DRGs is morphologically similar to DRG macrophages (compare arrowheads in Fig. 7D, H to Iba1^+^ macrophages in Fig. 9F, H). A CCL2–RFP signal was also in a membranous layer surrounding the DRG, which is most likely the meninges (Fig. 7). Macrophage and meningeal *Ccl2* could explain the residual expression in DCKO DRGs (Figs. 2, 7), but fibroblasts and endothelia can express *Ccl2* as well, and without co-labeling, we cannot rule out their contribution.

In another surprise, P0-Cre^+^ strains did not show a detectable decrease in DN *Ccl2* mRNA, suggesting Schwann cells contribute little to *Ccl2* expression. We confirmed this using CCL2–RFP animals. In these animals, most CCL2 expressing cells were macrophages, and there was no detectable decrease in CCL2 expressing cells in the DCKOs. This also implies that Schwann cells produce a relatively small amount of CCL2 and shows macrophages are the primary CCL2 source. This agrees with some of the earliest speculation about the source of DN CCL2 [20]. However, some F4/80^−^ CCL2–RFP^+^ cells resembled Schwann cells, and other studies have shown they can express CCL2 in culture, so they cannot be ruled out as a source [48, 49]. We also observed F4/80^−^ CCL2–RFP^+^ cells in the DCKOs without the typical “cigar-shaped” nucleus of Schwann cells, which implies a third cell type, possibly fibroblasts, expresses CCL2. Finally, there was strong CCL2 expression in the epi- and perineurium localized to macrophages and epithelial cells (upper nerve borders in Fig. 8D, F, H) which could also contribute to macrophage recruitment. These three alternative CCL2 sources explain the persistent *Ccl2* mRNA in all CKOs.

We next addressed the important claim made in Kwon et al., [21] that CCL2 is absolutely required for macrophage accumulation and macrophage induced enhancement of axon regeneration. We first expected that since *Ccl2* was substantially decreased in the DCKO DRGs, they at least would have impaired macrophage recruitment and subsequently a loss of CL-enhanced regeneration. However, in all CKOs macrophage accumulation was unaffected, and macrophages appeared functionally normally as there were no defects in myelin clearance or regeneration. We further examined CCL2 in the CL response by repeating these experiments in global *Ccl2* KO animals. Surprisingly, and in direct conflict with Kwon et al., [21], macrophage recruitment to the DRG and DN was unaffected in the KOs. The experiments from Kwon et al., [21] were replicated using the same *Ccl2* KO strain from Jackson, two macrophage labels, and the timepoint when they observed the largest difference between genotypes. With such similar methods, we cannot explain this discrepancy. Since CCL2 may help induce a pro-regenerative macrophage phenotype, we also tested regeneration and myelin clearance in the *Ccl2* KOs. Again, we found regeneration, CL-enhanced regeneration, and myelin clearance were unaffected in *Ccl2* KOs. Together, this shows that CCL2 is not required for macrophage recruitment or activation after a peripheral nerve injury and that these macrophages have no deficits in their ability to assist in myelin clearance and peripheral nerve regeneration in vivo. It remains possible that the phenotype and gene expression profile of these macrophages is altered in the absence of CCL2 (e.g., [50]). However, our results indicate that whatever changes occur they do not affect regeneration.

While we did not observe any deficits in macrophages using our peripheral nervous system injury model in *Ccl2* KO mice, full recovery after a nerve transection requires that the gap between the proximal and distal nerves be closed [51]. For small gaps, an endogenous bridge can form and for larger gaps, nerve grafts or artificial bridges can be used to allow for regeneration into the distal nerve. In both cases, macrophages are critical due in large part to their ability to stimulate angiogenesis and revascularization [52]. Bridge formation is also a length dependent process. A gap of about 3.5 mm or larger is thought to be the “critical gap” in that the proximal and distal stumps are unable to reconnect even after 14 days [53, 54]. Our transection model intentionally creates a large nerve gap and, it is important to note, that we do not observe any bridge formation even across the 9-day-old CL transections in WT animals. We do this so that axons do not reach the distal nerve and by their presence alter the signaling environment of the DN and DRG. However, it also means that we could not directly evaluate the function of CCL2/CCR2 signaling in bridge formation. In our experiments, we examined nerve regeneration from the site where the sciatic nerve was crushed (Fig. 1A). In the experiments of Pan et al. [50], in an effort to mirror the situation often faced by a neurosurgeon, they transected the sciatic nerve and introduced an acellular nerve graft to bridge the resulting gap. They found that macrophage recruitment into the graft was significantly reduced in *Ccl2* KO animals, as was angiogenesis and Schwann cell migration. Interestingly, NF200+ axon entry into the allograft was not reduced in *Ccl2* KO mice when examined 2 or 6 weeks after an injury, but functional recovery was inhibited at 6 week post-injury. Based on work by Cattin et al. [55], hypoxia is the initial signal that recruits macrophages and fibroblasts into the gap between nerves. In addition, as this gap is completely acellular, it follows that there would be nothing to produce CCL2 to recruit macrophages initially. As we have shown, macrophages in the nerve produce CCL2 (Fig. 8), and thus, it is likely that they could be the source of CCL2 for recruiting additional macrophages to a nerve bridge or allograft. Thus, while we did not observe any deficits in macrophages in *Ccl2* KO animals in the DRG or DN in our injury paradigm, it seems that CCL2 is involved after nerve transection when a nerve gap must be repaired.

A role for CCL2 in promoting CNS regeneration is still possible. Our assays tested CCL2 function in vivo in the PNS and demonstrated that CCL2 is not required for physiologic macrophage recruitment or regeneration. However, many previous regeneration assays have been done with DRGs in vitro or ex vivo, or in vivo in the CNS. Virally induced *Ccl2* expression in sensory neurons can recruit and activate macrophages in the DRG leading to enhanced regeneration in in vitro, ex vivo, or in vivo CNS assays [12, 21, 56, 57]. Culture systems lack many non-neuronal cells (i.e. macrophages, Schwann cells, etc.) and the close proximity they share with sensory neuron cell bodies in vivo. The CNS is also a more hostile environment and thus both systems may allow for, or require, an additional macrophage factor to enhance regeneration, and this factor may require CCL2 signaling. If true, CL-enhanced regeneration in the PNS and CNS are separable biological processes. Two early studies on CL-enhanced regeneration found that either a peripheral cut or crush injury could stimulate enhanced peripheral nerve regeneration, but only a cut injury enhanced regeneration in the dorsal root [58, 59]. If the processes enhancing peripheral and central regeneration could be used in combination, it may be possible to further enhance regeneration in the PNS and CNS.

As we have shown, CCL2 is not required for macrophage recruitment or activation but its receptor, CCR2, seemed necessary [11, 12]. Thus, we measured the expression of other CCR2 chemokines which could compensate for CCL2. We found *Ccl7* and *Ccl12* are also upregulated by nerve injury, and, in another report, CCL8 protein was shown to increase [11]. The role of these other, lower affinity, CCR2 chemokines is varied in other inflammatory models. In most models, loss of CCL2 causes a substantial reduction in leukocyte recruitment or pathogen clearance, which makes our finding more surprising [18, 30]. However, T-cells also use CCR2 to traffic to inflamed and infected locations, where they are a major cytokine source [60, 61]. Thus, loss of CCL2 can have compounding effects by disrupting signaling between macrophages, T-cells, and other immune cells. In homeostatic monocyte emigration from bone marrow, CCR2 signals are required [62], and CCL2 contributes to emigration, but CCL7 is more potent and both chemokines act additively [63]. CCL2 and CCL7 act additively in some inflammatory models as well [64, 65]. Finally, CCL2 is not required in some infection models [30], nor is CCL2 required for macrophages to invade and clear thrombi [66], or invade adipose tissue in obesity [67]. Thus, other CCR2 chemokines could be sufficient for the nerve injury response.

For CCL2 signaling to be required for the macrophage response to nerve injury, CCR2 signaling must also be required. To confirm our *Ccl2* KO finding, we reexamined the role of CCR2 in nerve injury using CCR2^gfp^ KI/KO animals which allowed us to distinguish recruited macrophages from residents. In the DN, we found most macrophages are derived from the circulation, and CCR2 signaling is required for a maximal macrophage response. Interestingly, even though there is a marked reduction in recruited DN macrophages in CCR2^gfp^ KOs, they are not completely absent which implies an additional CCR2-independent recruitment mechanism. These recruited cells could be patrolling CX3CR1^hi^, CCR2^lo^ monocytes or a CCR5^+^, CCR2^+^ monocyte subset using CX3CR1 or CCR5, respectively [43, 68]. The presence of recruited macrophages implies neither CCR2 nor CCL2 are absolutely necessary for some DN macrophage recruitment. In addition, there is an expansion of resident macrophages [46, 69]. Since the DN macrophage response is unaltered in *Ccl2* KOs, both alternative recruitment mechanisms, and resident macrophages are compensating for the deficiency confirming CCL2 is not required.

In the DRG we observed no statistically significant deficit in the macrophage response in the CCR2^gfp^ KOs. This differs from our laboratory’s previous CCR2 KO study, which reported that a significant number of DRG macrophages are recruited via CCR2 [11]. On closer examination, the differences might not be so great. We observe here a small decrease (~ 10%) in injury induced macrophages in the CCR2^gfp^ KOs compared to controls and this corresponds to the small increase (~ 10%) in recruited macrophages we observe in het controls relative to their sham. Theoretically, both numbers represent the contribution of recruited macrophages to DRG inflammation, and because they are similar, we speculate that there may be a small population of monocytes recruited to the DRG after injury. Flow cytometry results from our previous study showed that there were fewer macrophages in the axotomized *Ccr2* KO relative to WT, but there was also always an increase in the KO relative to its sham control [11]. Depending on the markers used, the CCR2 KO had ~ 10 to 30% reduction in macrophages compared to WT. The small additional decrease in macrophages could be due to loss of macrophages in the meninges or nerve area of the DRG, which both were excluded from the analysis in the present paper. Using a parabiosis approach, Kalinski et al. [14] also reported that most injury-induced DRG macrophages are residents and only a small number are recruited from circulation. Since DRGs rely primarily on a resident macrophage response and their response is unaltered in both *Ccl2* KOs and CCR2^*gfp*^ KOs, it is likely that CCL2 and CCR2 are only responsible for a small portion of the DRG injury response.

## Conclusions

In summary, the present study showed that CCL2 is not required for macrophage accumulation or axon regeneration after a sciatic nerve injury. Knocking out *Ccl2* specifically in sensory neurons, Schwann cells, or both, or using a global *Ccl2* KO is not sufficient to reduce macrophage accumulation, myelin clearance, or axon regeneration following a sciatic nerve injury. We found CCL2 is expressed broadly in both the DRG and distal nerve after injury, and in addition to *Ccl2,* other CCR2 ligands, *Ccl7* and *Ccl12*, are highly expressed in both tissues. Finally, *Ccr2*^gfp^ knock-in/knock-out animals were used to differentiate resident and recruited macrophages in the injured tissues. We found a significant decrease in all macrophages in the distal sciatic nerve in CCR2^gfp^  KO mice compared to CCR2^gfp^ hets. Importantly, there were still some GFP^+^ recruited macrophages as well as a relative expansion of CD68^+^ GFP^−^ resident macrophages in the KOs. In the DRG, we saw a small but insignificant decrease in CD68^+^ macrophages between CCR2^Gfp^ KO mice compared to CCR2^Gfp^ hets and interestingly, most DRG macrophages seem to be GFP^−^ residents. The findings from the knock-in/knock-out mice suggest that in the absence of CCL2, other CCR2 chemokines, resident macrophage proliferation, and CCR2-independent monocyte recruitment can compensate and allow for normal macrophage accumulation. Recognition of the relative contribution of resident and infiltrating cells to the totality of injury-induced macrophage accumulation in the DRG and sciatic nerve and understanding the complexity of signaling for monocyte recruitment from the blood into the injured peripheral nervous system will greatly aid the study of the immune system’s reaction to and involvement in peripheral nerve injuries.

## Data Availability

The data sets used and/or analyzed during the current study are available from the corresponding author on reasonable request.

## Abbreviations

CL: Conditioning lesion
DRG: Dorsal root ganglia
qPCR: Quantitative polymerase chain reaction
CKO: Conditional knock out
KO: Knock out
DN: Distal nerve
KI: Knock in
RFP: Red fluorescent protein
DPI: Days post-injury
GFP: Green fluorescent protein

## References

[1] McQuarrie IG, Grafstein B (1973). Axon outgrowth enhanced by a previous nerve injury. Arch Neurol.

[2] Zigmond RE, Echevarria FD (2019). Macrophage biology in the peripheral nervous system after injury. Prog Neurobiol.

[3] Ramon y Cajal S. Degeneration and regeneration of the nervous system. New York: Oxford University Press; 1928.

[4] Shapouri-Moghaddam A, Mohammadian S, Vazini H, Taghadosi M, Esmaeili SA, Mardani F, Seifi B, Mohammadi A, Afshari JT, Sahebkar A (2018). Macrophage plasticity, polarization, and function in health and disease. J Cell Physiol.

[5] Locati M, Curtale G, Mantovani A (2020). Diversity, mechanisms, and significance of macrophage plasticity. Annu Rev Pathol.

[6] Barrette B, Hebert MA, Filali M, Lafortune K, Vallieres N, Gowing G, Julien JP, Lacroix S (2008). Requirement of myeloid cells for axon regeneration. J Neurosci.

[7] Lindholm D, Heumann R, Meyer M, Thoenen H (1987). Interleukin-1 regulates synthesis of nerve growth factor in non-neuronal cells of rat sciatic nerve. Nature.

[8] Schreiber RC, Shadiack AM, Bennett TA, Sedwick CE, Zigmond RE (1995). Changes in the macrophage population of the rat superior cervical ganglion after postganglionic nerve injury. J Neurobiol.

[9] Lu X, Richardson PM (1993). Responses of macrophages in rat dorsal root ganglia following peripheral nerve injury. J Neurocytol.

[10] Lu X, Richardson PM (1991). Inflammation near the nerve cell body enhances axonal regeneration. J Neurosci.

[11] Lindborg JA, Niemi JP, Howarth MA, Liu KW, Moore CZ, Mahajan D, Zigmond RE (2018). Molecular and cellular identification of the immune response in peripheral ganglia following nerve injury. J Neuroimmunol.

[12] Niemi JP, DeFrancesco-Lisowitz A, Roldan-Hernandez L, Lindborg JA, Mandell D, Zigmond RE (2013). A critical role for macrophages near axotomized neuronal cell bodies in stimulating nerve regeneration. J Neurosci.

[13] Siebert H, Sachse A, Kuziel WA, Maeda N, Bruck W (2000). The chemokine receptor CCR2 is involved in macrophage recruitment to the injured peripheral nervous system. J Neuroimmunol.

[14] Kalinski AL, Yoon C, Huffman LD, Duncker PC, Kohen R, Passino R, Hafner H, Johnson C, Kawaguchi R, Carbajal KS (2020). Analysis of the immune response to sciatic nerve injury identifies efferocytosis as a key mechanism of nerve debridement. Elife.

[15] Bianconi V, Sahebkar A, Atkin SL, Pirro M (2018). The regulation and importance of monocyte chemoattractant protein-1. Curr Opin Hematol.

[16] Deshmane SL, Kremlev S, Amini S, Sawaya BE (2009). Monocyte chemoattractant protein-1 (MCP-1): an overview. J Interferon Cytokine Res.

[17] Semple BD, Kossmann T, Morganti-Kossmann MC (2010). Role of chemokines in CNS health and pathology: a focus on the CCL2/CCR2 and CXCL8/CXCR2 networks. J Cereb Blood Flow Metab.

[18] Shi C, Pamer EG (2011). Monocyte recruitment during infection and inflammation. Nat Rev Immunol.

[19] Tanaka T, Minami M, Nakagawa T, Satoh M (2004). Enhanced production of monocyte chemoattractant protein-1 in the dorsal root ganglia in a rat model of neuropathic pain: possible involvement in the development of neuropathic pain. Neurosci Res.

[20] Carroll SL, Frohnert PW (1998). Expression of JE (monocyte chemoattractant protein-1) is induced by sciatic axotomy in wild type rodents but not in C57BL/Wld(s) mice. J Neuropathol Exp Neurol.

[21] Kwon MJ, Shin HY, Cui Y, Kim H, Thi AH, Choi JY, Kim EY, Hwang DH, Kim BG (2015). CCL2 mediates neuron-macrophage interactions to drive proregenerative macrophage activation following preconditioning injury. J Neurosci.

[22] Zhang H, Boyette-Davis JA, Kosturakis AK, Li Y, Yoon SY, Walters ET, Dougherty PM (2013). Induction of monocyte chemoattractant protein-1 (MCP-1) and its receptor CCR2 in primary sensory neurons contributes to paclitaxel-induced peripheral neuropathy. J Pain.

[23] White FA, Feldman P, Miller RJ (2009). Chemokine signaling and the management of neuropathic pain. Mol Interv.

[24] Toews AD, Barrett C, Morell P (1998). Monocyte chemoattractant protein 1 is responsible for macrophage recruitment following injury to sciatic nerve. J Neurosci Res.

[25] Groh J, Heinl K, Kohl B, Wessig C, Greeske J, Fischer S, Martini R (2010). Attenuation of MCP-1/CCL2 expression ameliorates neuropathy in a mouse model for Charcot-Marie-Tooth 1X. Hum Mol Genet.

[26] Zurborg S, Piszczek A, Martinez C, Hublitz P, Al Banchaabouchi M, Moreira P, Perlas E, Heppenstall PA (2011). Generation and characterization of an Advillin-Cre driver mouse line. Mol Pain.

[27] Feltri ML, D'Antonio M, Previtali S, Fasolini M, Messing A, Wrabetz L (1999). P0-Cre transgenic mice for inactivation of adhesion molecules in Schwann cells. Ann N Y Acad Sci.

[28] Ge S, Murugesan N, Pachter JS (2009). Astrocyte- and endothelial-targeted CCL2 conditional knockout mice: critical tools for studying the pathogenesis of neuroinflammation. J Mol Neurosci.

[29] Shi C, Jia T, Mendez-Ferrer S, Hohl TM, Serbina NV, Lipuma L, Leiner I, Li MO, Frenette PS, Pamer EG (2011). Bone marrow mesenchymal stem and progenitor cells induce monocyte emigration in response to circulating toll-like receptor ligands. Immunity.

[30] Lu B, Rutledge BJ, Gu L, Fiorillo J, Lukacs NW, Kunkel SL, North R, Gerard C, Rollins BJ (1998). Abnormalities in monocyte recruitment and cytokine expression in monocyte chemoattractant protein 1-deficient mice. J Exp Med.

[31] Satpathy AT, Briseño CG, Lee JS, Ng D, Manieri NA, Kc W, Wu X, Thomas SR, Lee WL, Turkoz M (2013). Notch2-dependent classical dendritic cells orchestrate intestinal immunity to attaching- and-effacing bacterial pathogens. Nat Immunol.

[32] Subang MC, Richardson PM (2001). Influence of injury and cytokines on synthesis of monocyte chemoattractant protein-1 mRNA in peripheral nervous tissue. Eur J Neurosci.

[33] Nadeau S, Filali M, Zhang J, Kerr BJ, Rivest S, Soulet D, Iwakura Y, de Rivero Vaccari JP, Keane RW, Lacroix S (2011). Functional recovery after peripheral nerve injury is dependent on the pro-inflammatory cytokines IL-1beta and TNF: implications for neuropathic pain. J Neurosci.

[34] Schreiber RC, Krivacic K, Kirby B, Vaccariello SA, Wei T, Ransohoff RM, Zigmond RE (2001). Monocyte chemoattractant protein (MCP)-1 is rapidly expressed by sympathetic ganglion neurons following axonal injury. NeuroReport.

[35] Yoshimura T, Yuhki N, Moore SK, Appella E, Lerman MI, Leonard EJ (1989). Human monocyte chemoattractant protein-1 (MCP-1). Full-length cDNA cloning, expression in mitogen-stimulated blood mononuclear leukocytes, and sequence similarity to mouse competence gene JE. FEBS Lett.

[36] Lindborg JA, Mack M, Zigmond RE (2017). Neutrophils are critical for myelin removal in a peripheral nerve injury model of Wallerian degeneration. J Neurosci.

[37] Boring L, Gosling J, Chensue SW, Kunkel SL, Farese RV, Broxmeyer HE, Charo IF (1997). Impaired monocyte migration and reduced type 1 (Th1) cytokine responses in C-C chemokine receptor 2 knockout mice. J Clin Investig.

[38] Sarafi MN, Garcia-Zepeda EA, MacLean JA, Charo IF, Luster AD (1997). Murine monocyte chemoattractant protein (MCP)-5: a novel CC chemokine that is a structural and functional homologue of human MCP-1. J Exp Med.

[39] Franci C, Wong LM, Van Damme J, Proost P, Charo IF (1995). Monocyte chemoattractant protein-3, but not monocyte chemoattractant protein-2, is a functional ligand of the human monocyte chemoattractant protein-1 receptor. J Immunol.

[40] Zhao H, Duan LJ, Sun QL, Gao YS, Yang YD, Tang XS, Zhao DY, Xiong Y, Hu ZG, Li CH (2020). Identification of key pathways and genes in L4 dorsal root ganglion (DRG) after sciatic nerve injury via micoarray analysis. J Investig Surg.

[41] Wang Q, Zhang S, Liu T, Wang H, Liu K, Wang Q, Zeng W (2018). Sarm1/Myd88-5 regulates neuronal intrinsic immune response to traumatic axonal injuries. Cell Rep.

[42] Popiolek-Barczyk K, Ciechanowska A, Ciapała K, Pawlik K, Oggioni M, Mercurio D, De Simoni MG, Mika J (2020). The CCL2/CCL7/CCL12/CCR2 pathway is substantially and persistently upregulated in mice after traumatic brain injury, and CCL2 modulates the complement system in microglia. Mol Cell Probes.

[43] Tacke F, Randolph GJ (2006). Migratory fate and differentiation of blood monocyte subsets. Immunobiology.

[44] Ginhoux F, Jung S (2014). Monocytes and macrophages: developmental pathways and tissue homeostasis. Nat Rev Immunol.

[45] Zhang L, Xie W, Zhang J, Shanahan H, Tonello R, Lee SH, Strong JA, Berta T, Zhang JM (2021). Key role of CCR2-expressing macrophages in a mouse model of low back pain and radiculopathy. Brain Behav Immun.

[46] Mueller M, Leonhard C, Wacker K, Ringelstein EB, Okabe M, Hickey WF, Kiefer R (2003). Macrophage response to peripheral nerve injury: the quantitative contribution of resident and hematogenous macrophages. Lab Investig.

[47] Muller M, Leonhard C, Krauthausen M, Wacker K, Kiefer R (2010). On the longevity of resident endoneurial macrophages in the peripheral nervous system: a study of physiological macrophage turnover in bone marrow chimeric mice. J Peripher Nerv Syst.

[48] Tofaris GK, Patterson PH, Jessen KR, Mirsky R (2002). Denervated Schwann cells attract macrophages by secretion of leukemia inhibitory factor (LIF) and monocyte chemoattractant protein-1 in a process regulated by interleukin-6 and LIF. J Neurosci.

[49] Subang MC, Richardson PM (1999). Tumor necrosis factor-alpha induces monocyte chemoattractant protein-1 mRNA in a Schwann cell line. Ann N Y Acad Sci.

[50] Pan D, Acevedo-Cintrón JA, Sayanagi J, Snyder-Warwick AK, Mackinnon SE, Wood MD (2020). The CCL2/CCR2 axis is critical to recruiting macrophages into acellular nerve allograft bridging a nerve gap to promote angiogenesis and regeneration. Exp Neurol.

[51] Zochodne DW (2012). The challenges and beauty of peripheral nerve regrowth. J Peripher Nerv Syst.

[52] Cattin AL, Lloyd AC (2016). The multicellular complexity of peripheral nerve regeneration. Curr Opin Neurobiol.

[53] Min Q, Parkinson DB, Dun XP (2021). Migrating Schwann cells direct axon regeneration within the peripheral nerve bridge. Glia.

[54] Pan D, Hunter DA, Schellhardt L, Jo S, Santosa KB, Larson EL, Fuchs AG, Snyder-Warwick AK, Mackinnon SE, Wood MD (2019). The accumulation of T cells within acellular nerve allografts is length-dependent and critical for nerve regeneration. Exp Neurol.

[55] Cattin AL, Burden JJ, Van Emmenis L, Mackenzie FE, Hoving JJ, Garcia Calavia N, Guo Y, McLaughlin M, Rosenberg LH, Quereda V (2015). Macrophage-induced blood vessels guide Schwann cell-mediated regeneration of peripheral nerves. Cell.

[56] Niemi JP, DeFrancesco-Lisowitz A, Cregg JM, Howarth M, Zigmond RE (2016). Overexpression of the monocyte chemokine CCL2 in dorsal root ganglion neurons causes a conditioning-like increase in neurite outgrowth and does so via a STAT3 dependent mechanism. Exp Neurol.

[57] Kwon MJ, Kim J, Shin H, Jeong SR, Kang YM, Choi JY, Hwang DH, Kim BG (2013). Contribution of macrophages to enhanced regenerative capacity of dorsal root ganglia sensory neurons by conditioning injury. J Neurosci.

[58] Oblinger MM, Lasek RJ (1984). A conditioning lesion of the peripheral axons of dorsal root ganglion cells accelerates regeneration of only their peripheral axons. J Neurosci.

[59] Richardson PM, Issa VM (1984). Peripheral injury enhances central regeneration of primary sensory neurones. Nature.

[60] Yopp AC, Fu S, Honig SM, Randolph GJ, Ding Y, Krieger NR, Bromberg JS (2004). FTY720-enhanced T cell homing is dependent on CCR2, CCR5, CCR7, and CXCR4: evidence for distinct chemokine compartments. J Immunol.

[61] Owen J, Punt J, Stranford S (2013). Kuby immunology.

[62] Serbina NV, Pamer EG (2006). Monocyte emigration from bone marrow during bacterial infection requires signals mediated by chemokine receptor CCR2. Nat Immunol.

[63] Tsou CL, Peters W, Si Y, Slaymaker S, Aslanian AM, Weisberg SP, Mack M, Charo IF (2007). Critical roles for CCR2 and MCP-3 in monocyte mobilization from bone marrow and recruitment to inflammatory sites. J Clin Investig.

[64] Jia T, Serbina NV, Brandl K, Zhong MX, Leiner IM, Charo IF, Pamer EG (2008). Additive roles for MCP-1 and MCP-3 in CCR2-mediated recruitment of inflammatory monocytes during *Listeria monocytogenes* infection. J Immunol.

[65] Mercer PF, Williams AE, Scotton CJ, José RJ, Sulikowski M, Moffatt JD, Murray LA, Chambers RC (2014). Proteinase-activated receptor-1, CCL2, and CCL7 regulate acute neutrophilic lung inflammation. Am J Respir Cell Mol Biol.

[66] Ali T, Humphries J, Burnand K, Sawyer B, Bursill C, Channon K, Greaves D, Rollins B, Charo IF, Smith A (2006). Monocyte recruitment in venous thrombus resolution. J Vasc Surg.

[67] Inouye KE, Shi H, Howard JK, Daly CH, Lord GM, Rollins BJ, Flier JS (2007). Absence of CC chemokine ligand 2 does not limit obesity-associated infiltration of macrophages into adipose tissue. Diabetes.

[68] Ginhoux F, Guilliams M (2016). Tissue-resident macrophage ontogeny and homeostasis. Immunity.

[69] Mueller M, Wacker K, Ringelstein EB, Hickey WF, Imai Y, Kiefer R (2001). Rapid response of identified resident endoneurial macrophages to nerve injury. Am J Pathol.

